# Extracting Multiscale Pattern Information of fMRI Based Functional Brain Connectivity with Application on Classification of Autism Spectrum Disorders

**DOI:** 10.1371/journal.pone.0045502

**Published:** 2012-10-08

**Authors:** Hui Wang, Chen Chen, Hsieh Fushing

**Affiliations:** Department of Statistics, University of California Davis, Davis, California, United States of America; Wake Forest School of Medicine, United States of America

## Abstract

We employed a multi-scale clustering methodology known as “data cloud geometry” to extract functional connectivity patterns derived from functional magnetic resonance imaging (fMRI) protocol. The method was applied to correlation matrices of 106 regions of interest (ROIs) in 29 individuals with autism spectrum disorders (ASD), and 29 individuals with typical development (TD) while they completed a cognitive control task. Connectivity clustering geometry was examined at both “fine” and “coarse” scales. At the coarse scale, the connectivity clustering geometry produced 10 valid clusters with a coherent relationship to neural anatomy. A supervised learning algorithm employed fine scale information about clustering motif configurations and prevalence, and coarse scale information about intra- and inter-regional connectivity; the algorithm correctly classified ASD and TD participants with sensitivity of 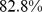 and specificity of 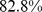. Most of the predictive power of the logistic regression model resided at the level of the fine-scale clustering geometry, suggesting that cellular versus systems level disturbances are more prominent in individuals with ASD. This article provides validation for this multi-scale geometric approach to extracting brain functional connectivity pattern information and for its use in classification of ASD.

## Introduction

Recent joint advances in functional magnetic resonance imaging (fMRI) technology and in graph theoretical analysis have allowed neuroscientists to devote great amount of research attentions and efforts on constructing complex networks of functional brain connectivity [1]. The ultimate goal is to understand how the brain works. This understanding can be facilitated by discovering both macro- and micro-scopic connectivity patterns that bear future diagnostic tools for brain related disorders like autism spectrum disorders (ASD), for example. This research direction has become one of the major trends in neurosciences [1]. Hence developing effective methodology for network analysis that reliably extracts informative patterns of functional brain connectivity will be the focus of the paper.

However there are several challenges facing scientific endeavors for functional brain connectivity patterns and their clinical associations. One of the challenges is the complexity due to clinical heterogeneity. For instance ASD are heterogeneous neuro-developmental disorders that involve diverse neuropathology [2]. Perhaps the most consistent finding about ASD is that they are disorders of functional connectivity [3]–[5]. It is natural to ask what typical ASD connectivity patterns may be identified? Currently there are few coherent arguments (whether empirical or theoretical) on the status of short- and long-range vs. over- or under-connectivity reported in literature [6]–[10]. Such paucity of explanation points to the need for cohesive framework for accurately describing the distribution and network connectivity pertinent to abnormalities [11].

The pattern extraction methodology presented here contributes to the necessary conditions to establish this type of cohesive framework. They are necessary conditions because pattern information is practical and instrumental for devising classification or diagnostic tests [5], [12]. The case of ASD illustrates the computational challenges that we wish to address by means of a supervised learning rule. When analyzing functional connectivity under both resting state and task related neural activity [1], [13], [14], local regional data of time series from brain scans are used to construct a correlation matrix, known as a functional brain graph. This is typically followed by some form of “thresholding” on the matrix to identify significant connections. Finally, topological properties of these may be investigated with graph theoretical metrics that describe networks including those assessing small-worldness [1] and community structure [15].

Several methodological difficulties are evident with these approaches and others related to thresholding and prescribing of network topology based on correlation matrices. The problem of thresholding is complex given multiple scales being present in most networks and the consequent need to correct for multiple comparisons. And thresholding is a type of truncation that results in information loss [1]. Another critical problem, which is typically unnoticed, is that results with different scales across participants are produced, and then potentially distorted information is summarized. There are three immediate scenarios that propagate local information distortion to global errors. First, a slightly perturbed correlation matrix might fail to retain the original relational structure among ROIs. Second, many connectivity clusters of ROIs could have been truncated due to their heterogeneous connectivity scales. Third, the geometry of several clusters merging into a larger conglomerate would be completely missed.

It is reasonable to assume that solutions to these computational challenges will facilitate translational medical outcomes for diseases like ASD. Specifically, we demonstrate how to effectively extract multiscale pattern information of functional brain connectivity from fMRI based correlation matrix data. We then construct a learning algorithm for classification of ASD. The key computational methodology employed here is the data cloud geometry algorithm developed by Fushing and McAssey [16]. Each subject-specific correlation matrix is converted into connectivity clustering geometry built according to a sequence of critical scales. In a hierarchy format, each level of such geometry is a clustering configuration partitioning 106 ROIs into subsets of ROIs being close or similar to each other on a relevant scale. In contrast, ROIs that are not similar with respect to the scale are parsed into separate clusters. All computations for cluster membership and cluster number per level are data-driven. The number of clusters at each scale is determined by an eigenvalue plot derived from the normalized graph Laplacian of a computed matrix of cluster-sharing probability. Then cluster memberships are extracted accordingly. The bottom level of the hierarchy corresponds to a fine scale and consists of many small clusters (2 or 3 ROIs each). As the scale gets larger, small clusters close to each others on the lower level merge, which leads to larger and fewer clusters. The top of the hierarchy consists of a single cluster.

In data cloud geometry algorithmic computations, this scale-sensitive matrix of cluster-sharing probability summarizes the result of regulated Markovian mechanisms, that reflect the “distance” between ROIs as well as its clusters, as would be clear in *Materials and Methods* Section. But very briefly, a scale determines the closeness between any two ROIs, so a Markov chain can be built with higher probability to move from one ROI to its closer neighbors than to one far apart. Then this Markov chain would tend to go around and tentatively be trapped within a cluster. Therefore one clustering configuration pertaining to a scale could be found based on a large collection of specially designed Markov chains, so-called regulated Markov random walks, which are capable of exhaustively exploring the whole connectivity among all ROIs; and different scales could bring out significantly different clustering configuration. Collectively we find a hierarchy of such configurations and call it a connectivity clustering geometry. This is the basic idea of data cloud geometry. Since this hierarchy is constructed using one individual's correlation matrix, it bears a geometric structure that is specific to this individual. Though the sequences of critical scales used for connectivity clustering geometries vary from individual to individual, the collection of connectivity clustering geometries constructed as such from the participants are compatible in the sense that we individually tune to a “right scale” for a common clustering structure across all participants. This is similar to what is done when focusing a microscope. Since the focus is on functional connectivity, the “tuning” produces more or less the same number of clusters across participants in the data set. It is our contention that when individuals are tuned to the same clustering structure, characteristics of their functional connectivity can be most readily and legitimately compared.

For each participant in ASD group and TD group, two separate multi-scale connectivity clustering geometries are derived via data cloud geometry based on each individual's fMRI-based correlation matrix constructed from the 106 ROIs during red and green trials in the cognitive control task, respectively. A 3-D functional connectivity network is built based on each multi-scale geometry of each individual subject. Network representation of such geometries might be taken as subject's pseudo-neuroanatomy, so they could be compared based on their differences in network patterns for classification and other purposes. Here, by looking at one fine and one coarse scale level of connectivity clustering geometry, we demonstrate how to devise a supervised learning algorithm for classifying members between ASD group and TD group.

## Materials and Methods

### Data Description

**Figure 1:**
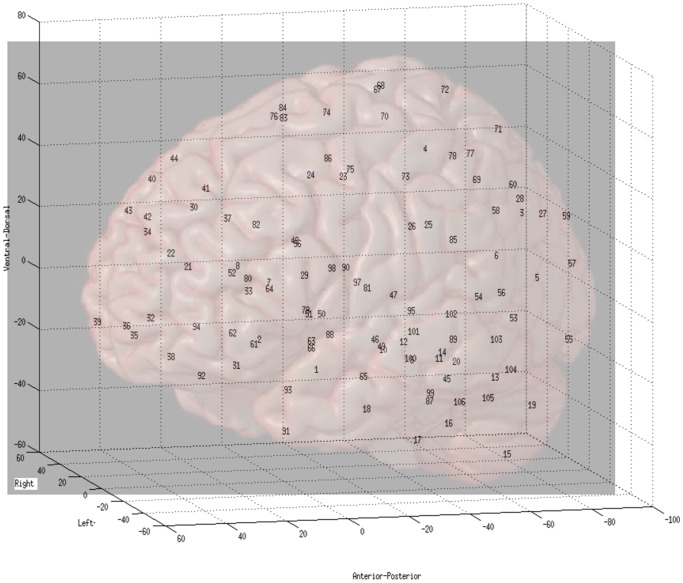
106 ROIs' 3-D locations on brain regions.

Participants included 58 adolescent subjects: 29 with ASD, and the other 29 typical developing (TD) individuals. Participants with ASD were assessed with standard measures. All these individuals participated in the study of Solomon et al [10], and measures were described therein. The study included 5 female subjects in each group to approximate the male to female gender ratio in the population of individuals with autism. Functional time series from 106 ROIs were collected during both red and green trials of the Preparing to Overcome Prepotency Task (POP; [17]–[20]). The regions were defined using the aal atlas [21], which parses the brain into 116 regions; this 116-region partition has been used in both task-related functional MRI studies and resting-state functional MRI studies [22]. 10 regions were removed from the analysis due to coverage issues (noisy signal). The 106 ROIs' 3-D locations are shown in Fig. 1 for reference convenience. Also see supplementary file S1 for the names of the 106 ROIs and their 3-D coordinates. Two 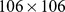 beta-series correlation [23] matrices are derived corresponding to the two trials from each participant. To calculate correlation between ROIs, beta-series correlation method is employed, which is different from the time series method. For this method, a generalized linear model (GLM) was constructed to include every stage of every trial with a separate covariate to obtain trial-to-trial parameter estimates of stage-specific activity. All estimated parameter values from each trial were sorted according to task stage into sets of condition specific betas, i.e., beta series, and correlated across brain regions. [10]. Each of this beta-series correlation matrix is the basis for subject and trial specific functional brain connectivity.

### Data Cloud Geometry

In this section we will illustrate how to obtain connectivity clustering geometry from a similarity measure via data cloud geometry algorithm proposed by Fushing and McAssey [16]. Then in the following section *Temperature Tuning* we will schematically present the connectivity clustering geometry via a cluster-sharing probability matrix, and display part of this geometric structure through a 3-D network of functional brain connectivity. At the last we will show an overall view of a coherent relationship among all participants' geometries of functional brain connectivity.

The advantage of the algorithm is that the geometry of a data cloud could be computed on multiple scales without prior knowledge about its structure. The concepts of ÒtimeÓ and ÒtemperatureÓ are introduced to construct a hierarchical geometry based on local information provided by a similarity measure. Specifically, along the time axis, a regulated random walk incorporated with recurrence-time dynamics detects information about the number of clusters and the corresponding cluster membership of each node; along the temperature axis, a few temperatures corresponding to phase transitions will be utilized to build the geometric hierarchy of a data cloud. At each chosen temperature, a cluster-sharing probability matrix is constructed to summarize information extracted from a number of regulated random walks [16].

First a correlation matrix is used to construct a similarity matrix, i.e., the absolute value of its 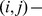
th entry is used as a similarity measure between the 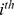 and 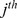 ROIs. Rigorously this measurement has an inherent and unknown initial scale 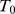 that may differ significantly among participants. Hence, we need to employ a scale-standardization analogous to the tuning resolution in the hypothetical microscope described in the introduction.

For the 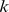-th subject, the correlation measurement between the pair of the 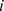-th and 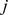-th ROIs is denoted as 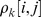. Its absolute value could be expressed as
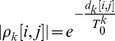
where 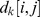 denotes the corresponding hypothetical underlying distance between the 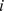-th and 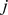-th ROIs with respect to the unknown initial scale 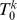.

Consider the following power transformation,
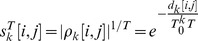
which results the similarity 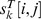 between the two ROIs under the power transform scale 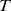. It is noted that the scale underlying this computable similarity is the unknown 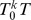. However we will apply a very wide range of 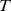 values in order to reveal the whole geometry of 106 ROIs embedded within the correlation matrix 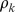. That is, the product 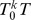 in the 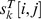 would take values in a wide range. Hence the unknown 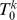 parameter becomes irrelevant in the whole process of constructing connectivity clustering geometry. Here we denote the collection of similarity matrix as 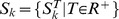.

For each chosen temperature 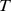, we applied the data cloud geometry algorithm illustrated above to construct the cluster-sharing probability matrix. The information of number of clusters is typically obtained through an eigenvalue plot of the normalized graph Laplacian of the cluster-sharing probability matrix. The number of significantly non-zero normalized eigenvalues is taken to be the number of clusters. Information about clustering membership is acquired from pruning the hierarchical clustering (HC) tree [24], which is constructed based on the cluster-sharing probability matrix. Therefore, ROIs with high cluster-sharing probability would be clustered together, and ROIs with low cluster-sharing probability would be separated into different clusters. This clustering result is termed by the level of connectivity clustering geometry on the 106 ROIs at temperature 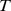.

#### Temperature Tuning

**Figure 2:**
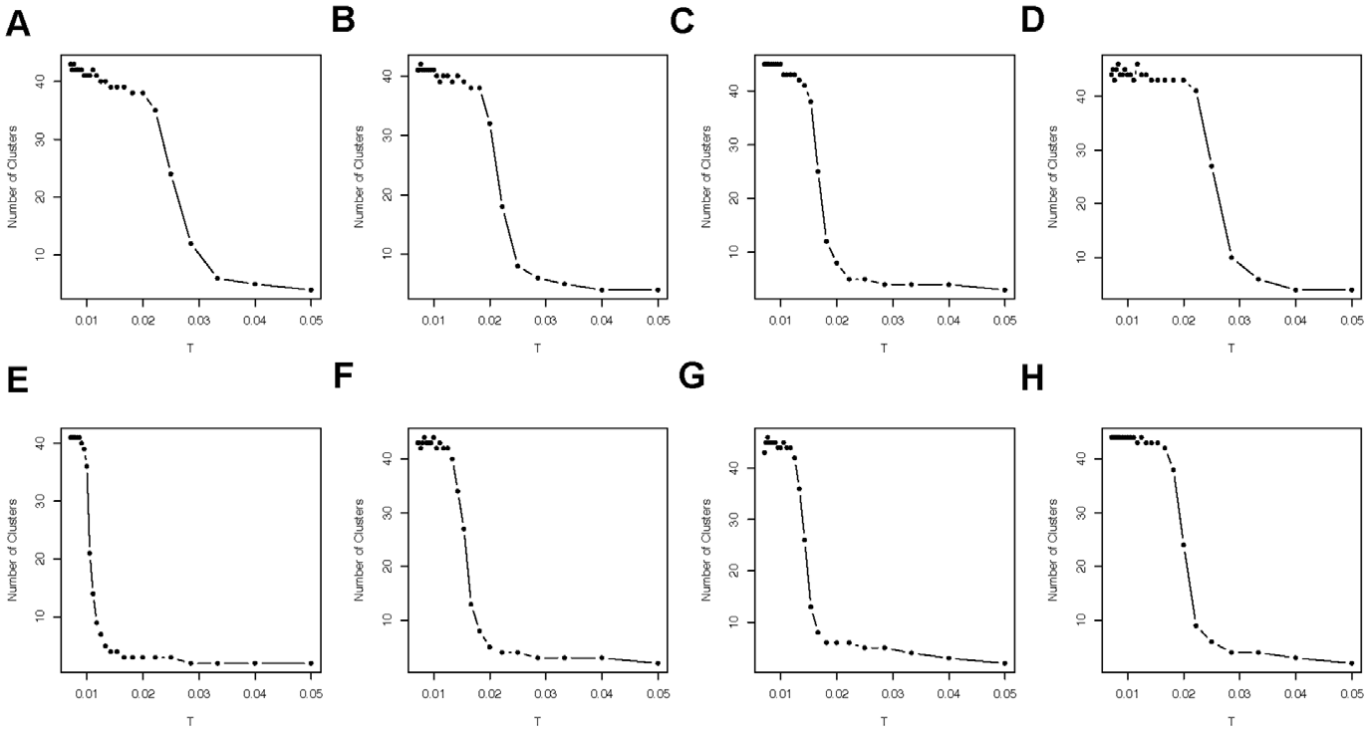
Eight individuals' evolution of number of clusters via eigenvalue plot. Top four panels for ASD subjects and bottom four panels for TD subjects in green trials.

By tuning the temperature 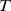, we observe that, when 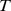 is very small, 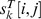 can be close to 0 for any correlation 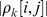 being only slightly less than 1; 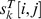 is significantly larger than 0 only when 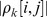 is very close to 1. That is, most similarity measurements are close to 0, except for those pairs which have very high correlation under the very small focal scale. This scale leads us to see many small core clusters. On the other end of extreme of the focal scale, when 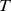 is very large, most similarity measurements are very close to 1. Here there would be only one cluster for all the nodes. Therefore we could visualize an evolution of clustering structures from fine to coarse with 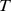 going from small to large. In fact this evolution is characterized by a sequence of critical temperatures where phase transitions of clustering geometry occur. This fact can be seen from the trajectory of number of clusters with respect to temperature in Fig. 2. The common pattern is as follows. When temperature is relatively low, the number of clusters remains high, then there is a steep drop and then the number of clusters slowly converges to 1 as temperature increases.

**Figure 3:**
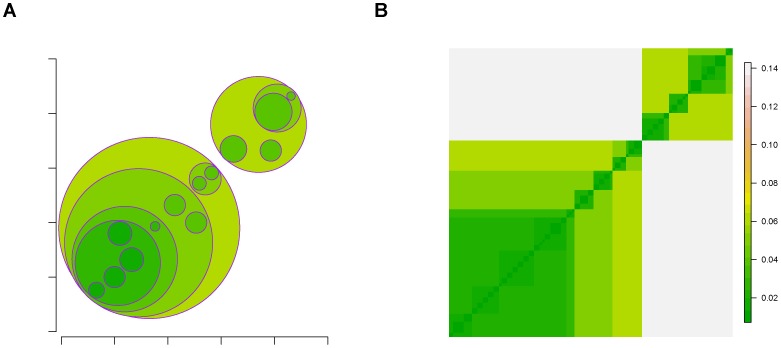
Schematic figure for final results of data cloud geometry applied on relational data. (A) Multiscale clustering geometry; (B) matrix representation.

The hierarchical levels of clustering configurations corresponding to this sequence of critical temperatures are jointly called connectivity clustering geometry. A schematic representation of such connectivity clustering geometry is given in Fig. 3. In Fig. 3(a), the small circles with highest color intensity correspond to clusters found at lowest temperature, so are the small squares with same color intensity on the matrix's diagonal in Fig. 3(b). As color gradually fades, the larger circles and larger squares are standing for conglomerated clusters by merging small clusters contained within. The vertical scale on the Fig. 3 (b) is the temperature scale.

**Figure 4:**
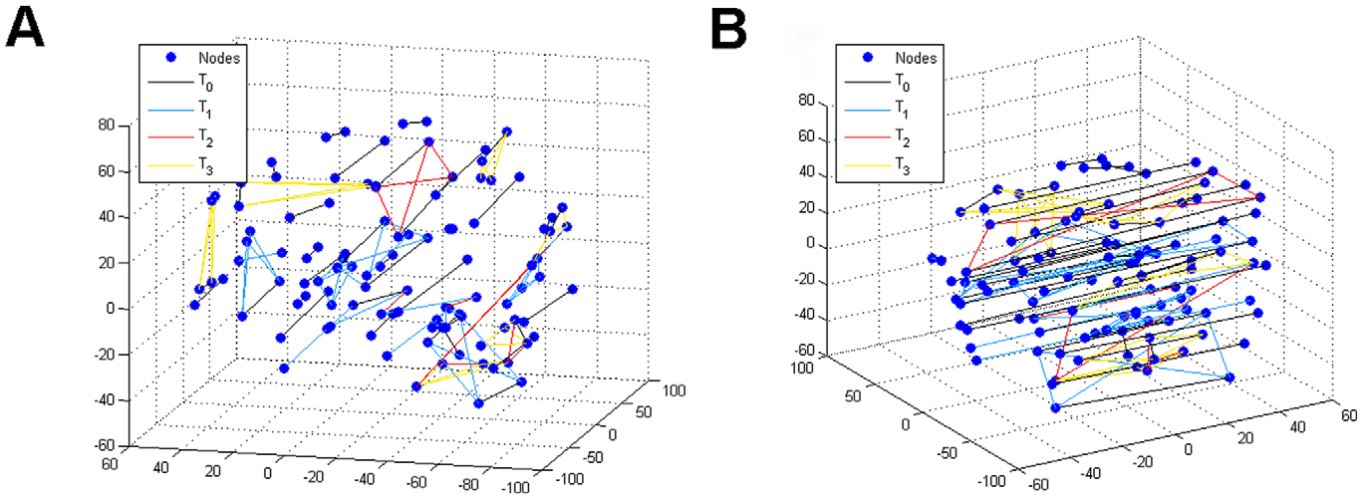
3-D functional brain connectivity. Patterns of network connectivity under four different temperatures. (A) Viewing from the same direction of the brain presented in Fig. 1; (B) Viewing from nearly back of a brain.

Another way to visualize such connectivity clustering geometry on 106 ROIs is to wire any pair of cluster-sharing ROIs on the brain's 3-D Euclidean coordinate. Fig. 4 (a) shows a network of connectivity viewing from the right brain hemisphere, while Fig. 4 (b) shows the same network viewing from the back of a brain. The latter view reveals evidently strong connectivity between ROIs corresponding located on the right and left brain hemispheres. This connectivity pattern is real because majority of ROIs are matched in a form of left-and-right correspondence pair.

**Figure 5:**
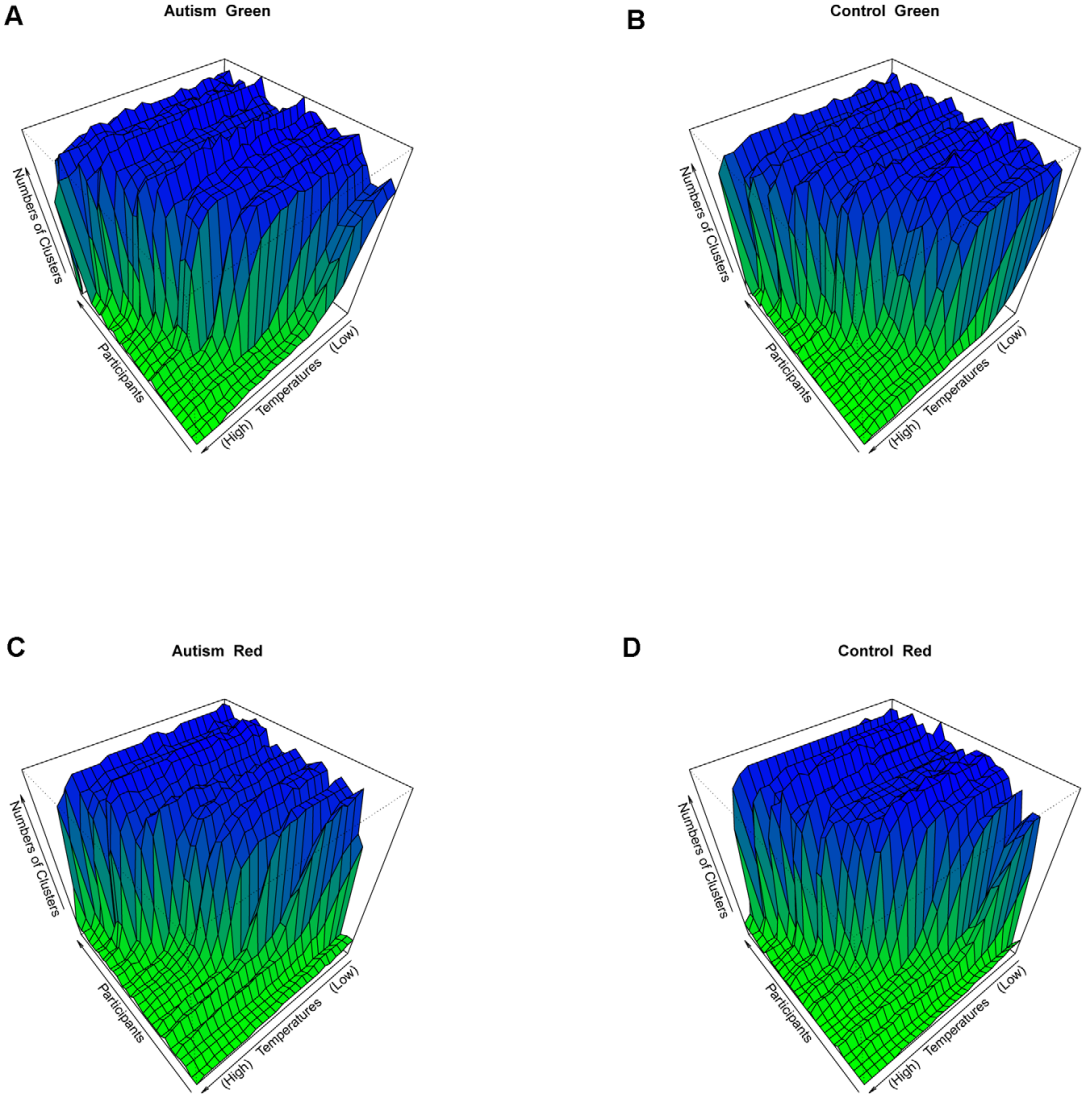
Group based multi-scales. The data are grouped into four by (ASD, TD)
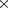
(Green, Red). The numbers of clusters are plotted against the temperatures for all the participants in each group. (A) is for ASD group when green trials were performed and (C) is for ASD group when red trials were performed. The right two panels are the plots for TD group, in which (B) is for green trials and (D) is for red trials.

The individual trajectories in Fig. 2 further reveal the important common multi-scale feature of data cloud geometry: there are initially more than 40 clusters. This number slowly decreases and then drops steeply to ten before slowly decreasing to one, in spite of the fact that their initial scale was unknown. 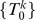 are apparently heterogeneous. To further confirm this common multi-scale feature, we compile the 29 trajectories together according to two different trial types and groups in Fig. 5. Together they reveal an overall view of a coherent clustering geometric relationship among all the participants' functional brain connectivity.

Fig. 5 shows overall strong evidence of the multi-scale clustering structure embedded within each correlation matrix with heterogeneous initial scales 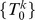. Hence it is essential to emphasize that any task involving comparing two or more correlation matrices needs to be performed on the base of matching clustering structures. Specifically two levels of connectivity clustering configurations on two different hierarchies respectively match when they both have the same number of clusters. This procedure is referred as “tuning” among different connectivity clustering geometries.

We further illustrate that such tuning is a necessary step in extracting comparable functional connectivity pattern information across subjects. For example, consider the scenario with T = 1, without making power transformations on all correlation matrices. Since T = 1 is way out of the range of horizontal 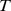 axis in Fig. 2 and 5, there would be only one cluster for all individual subjects. Hence, if a network such as that shown in Fig. 4 is constructed for each subject, each will have a complete graph since all ROIs are connected. However if a fixed number, more than one, of clusters are imposed on this scale, and any popular clustering approach, such as k-means or spectral clustering algorithm, is used, we would expect random partition which barely bears any useful information. If consider a scenario on the other extreme with a fixed small 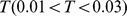 being used for all subjects, then all subjects will have rather heterogeneous numbers of clusters on the level of data cloud geometry. The heterogeneity would blur sensible pattern information for between-group comparison. By avoiding these two extreme scenarios, we demonstrate the merit of this tuning-to-right connectivity clustering geometry in the rest of this paper by developing an effective supervised learning based classification approach for ASD.

### Extracting function connectivity pattern information

Based on the two collections of 29 matrices 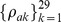 from ASD group and 29 matrices 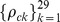 from TD group for both green and red trials, applications of data cloud geometry algorithm produce collections of subject-specific connectivity clustering geometry. In this paper we consider extracting pattern information from both fine and coarse scale connectivity clustering geometry for supervising learning purposes. The extraction of coarse scale pattern information is illustrated first, followed by that of fine scale pattern information.

#### Coarse scale pattern information

Autism is a disorder of diverse neuropathology, i.e., atypical functioning has been reported at a “coarse scale” across multiple brain regions, structures, and neural circuits [25]. For connectivity information pertaining to this scale, we employ an atlas of brain with 10 brain regions [21]. The distribution of the 106 ROIs on these 10 regions is reported in Table 1. Specifically, subcortical region is the collection of ROIs classified as subcortical structure, including amygdala nucleus, caudate nucleus, palladium, putamen and thalamus; central region refers to the collection of ROIs between the frontal lobe and the parietal lobe, including pre central gyrus, post central gyrus and rolandic operculum. With respect to this anatomic structure, we tune each of 58 participants' connectivity clustering geometries to the level with 10 clusters. We first address the question of whether this level of connectivity clustering geometry is consistent with the deterministic anatomical brain regions.

**Table 1:** Distribution of 106 ROIs on the brain atlas with 10 regions.

Anatomical Region	Subcortical	Parietal	Occipital	Cerebelum	Frontal	Temporal	Limbic	Insular	Central	Cingulum
Number of ROIs	10	10	14	20	22	8	8	2	6	6

**Figure 6:**
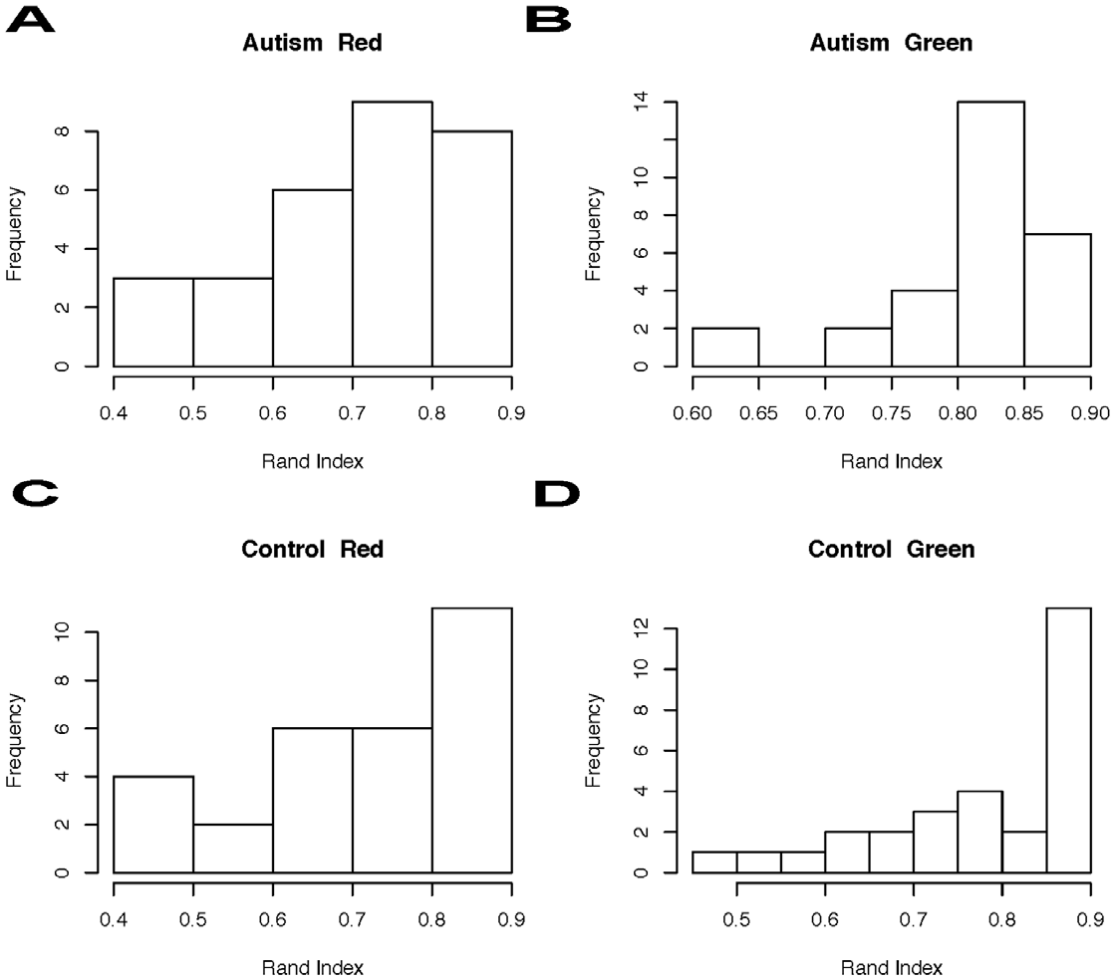
Rand index: tuned at 10-cluster-scale. For each group ((ASD, TD)
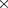
(Green, Red)), the histogram of the Rand index is given.

Firstly, we calculate a measure of concordance between these two grouping approaches on 106 ROIs for each individual via Rand index [26]. A Rand index is defined as the proportion of concordant grouping with respect to the two grouping on all pairs of ROIs, i.e.,
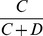
where C is the number of pairs with which both grouping methods reach concordant decisions: both determine the pair of ROIs being in the same group or in different groups; D is the number of pairs with which the two grouping methods reach different decisions. We report the Rand indices for the four categories: (ASD, TD)
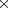
(Green-trial, Red-trial). The four histograms in Fig. 6 show that this 10-cluster level of connectivity clustering geometry has very high concordance with the anatomical structure.

This result of high concordance between the two ROIs grouping approaches strongly implies that our connectivity clustering geometry is not only realistic, but can also provide pattern information for constructing a supervised learning algorithm with potential power of classifying ASD memberships against TD's. The reasoning behind this heuristics is as follows. One intra-regional connectivity remaining as one intra-cluster connectivity is likely to be corresponding short-range connectivity, while one inter-regional connectivity remaining as one inter-cluster connectivity is likely to be corresponding long-range connectivity. If such correspondences are reasonable, then we can test whether there are connectivity pattern differences of such kinds existing between ASD and TD groups. Thus these testings are somehow checking all possible short- and long-range vs. under- or over-connectivity status across 10 brain regions. For extracting this coarse level pattern information, we compute the proportion of intra-connectivity within each anatomical region that remains as intra-connectivity under the connectivity clustering geometry, as well as the inter-connectivity between all possible 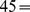 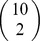 region pairs in each individual that remain. The computations result 55(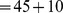, 45 inter-regional connectivity and 10 intra-regional connectivity) two-sample data sets: 29 measurements for ASD and control group each. Two-sample t-tests were performed on each two-sample data set to identify potentially abnormality on regional connectivity for ASD, which is taken as one piece of pattern information on the coarse level.

Upon red and green cognitive trials and among 55 multiple t-statistics, we report those testing results exceeding a 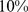 critical value in Table 2. We found 10 pieces of pattern information: 7 regional or inter-regional ones on the red trial and 3 on the green trial. A positive t-value in Table 2 means more connectivity in the autistic group, and a negative value indicates more connectivity in the control group.

**Table 2:** Significant differences in connectivity, t test statistic, and p-value.

Red			Green		
Connectivity	t-value	p-value	Connectivity	t-value	p-value
Within Parietal	1.85	0.070	Between Subcortical and Frontal	1.68	0.099
Between Cerebelum and Central	1.80	0.078	Between Parietal and Occipital	1.94	0.058
Between Temporal and Limbic	2.37	0.022	Within Occipital	−2.79	0.008
Within Insular	−1.67	0.101			
Between Subcortical and Occipital	2.05	0.046			
Between Limbic and Parietal	2.03	0.048			
Between Limbic and Occipital	1.79	0.080			

#### Fine scale pattern information

In this section we extract pattern information from a fine scale of connectivity clustering geometry. We consider the finest possible scale (T = 0.001) that corresponds to the bottom level of the hierarchy consisting of 49 or 50 clusters. This bottom level is selected primarily due to its robustness to temperature changes, as seen in Fig. 2 and 5. It has the potential to reveal the anatomical left-and-right relational patterns among the 106 ROIs.

To begin our formal development, each identified small core cluster in this bottom-level clustering configuration is called a motif, and the subject-specific collection of motifs is termed as a motif configuration. These motifs constitute the building blocks of clusters on upper levels of the geometry. It is similar with network motifs in [27]. And the concept of motif configurations is analogous to Pott's model of ferromagnetism in statistical mechanics where particles with the same spin state belong to the same cluster [28]. The ensemble of 29 individuals' motif configurations makes up one of 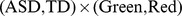 group-trial specific motif domain, which is characterized by its motif 's prevalence (the frequency of a motif in 29 subjects). The fine scale pattern information is to be extracted from the relationship between an individual motif configuration and a corresponding motif domain with motif prevalence.

**Figure 7:**
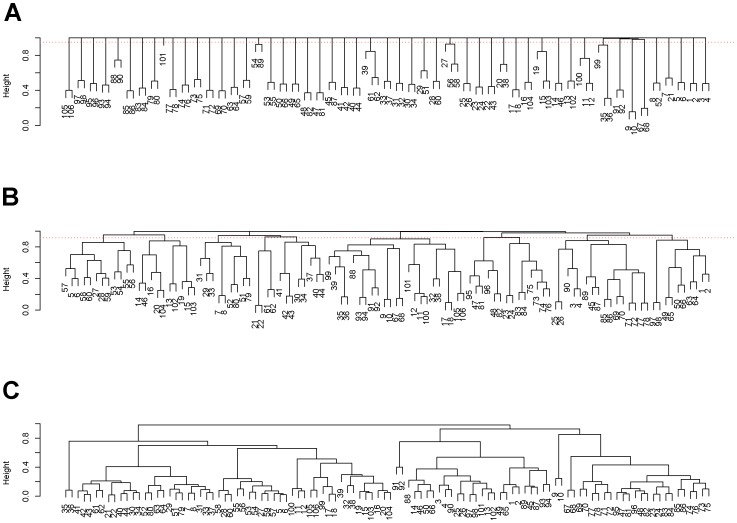
Three version of HC trees. (A) Fine scale HC-tree based on cluster-sharing probability matrix; (B) Coarse scale HC-tree based on cluster-sharing probability matrix; (C) HC-tree based on “raw” correlation matrix.

The detailed algorithmic computation for motif configuration and domain is given as follows. By tuning bottom-level connectivity clustering geometry, we identify a very low temperature with which the eigenvalues of the normalized graph Laplacian of the cluster-sharing probability matrix indicates around 50 clusters. We construct a hierarchical clustering (HC) algorithm (with complete module) based on this cluster-sharing probability matrix, as shown in Fig. 7(a). A range of threshold values on the vertical axis, 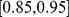, would prune the HC-clustering tree into nearly 50 branches. The average branches number is 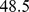 over all participants (at threshold 0.85). Here one branch is one motif. The set of motifs is a subject-trial specific motif configuration. We excluded singletons in the motif information considered here. We use the notation 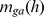 for the motif configuration of 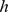-th ASD subject on green trials, and 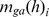 as its 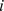-th motif element. And 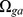 stands for the motif domain and 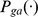 is defined as a motif prevalence function on this domain. Similar notations are used for red trials and for those pertaining to the TD group. Here we report a partial list of a motif domain 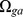 and values of the motif prevalence function 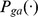 in Table 3 with 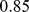 chosen as a threshold for pruning HC Trees. See supplementary file S3 for the HC trees of the 106 ROIs for all the 58 subjects in the green trials.

**Table 3:** Hierarchical Clustering Motifs Summarization.

motif	prevalence	prevalence	prevalence	motif	prevalence	prevalence	prevalence
	difference	(autism)	(control)		difference	(autism)	(control)
87 , 88	11	10	21	42 , 43	3	25	22
63 , 79	8	13	21	57 , 58	3	14	17
89 , 90	8	9	17	93 , 94	3	11	14
69 , 70	7	13	6	13 , 14	3	16	13
19 , 20	6	16	22	73 , 75	3	18	15
85 , 86	6	22	16	61 , 62	2	26	28
48 , 82	6	18	12	105 , 106	2	12	10
55 , 56	6	12	18	21 , 22	2	27	29
11 , 101	6	4	10	74 , 76	2	21	19
103 , 104	5	10	15	3 , 4	2	13	11
13 , 102	5	5	10	27 , 28	2	15	17
51 , 52	5	16	21	1 , 2	2	10	12
45 , 46	5	14	19	49 , 50	2	10	12
64 , 80	5	14	19	7 , 8	1	27	28
15 , 16	5	9	14	31 , 32	1	12	13
95 , 96	5	11	6	97 , 98	1	27	26
5 , 6	5	16	11	83 , 84	1	26	27
81 , 95	5	5	10	25 , 26	1	27	26
47 , 81	4	11	7	23 , 24	1	28	27
30 , 34	4	17	13	71 , 72	1	18	17
37 , 41	4	17	13	59 , 60	1	10	11
40 , 44	4	19	15	65 , 66	1	11	10
9 , 10	4	15	19	91 , 92	1	19	20
99 , 100	4	13	9	38 , 39	0	17	17
35 , 36	3	25	22	29 , 33	0	21	21
11 , 100	3	8	11	67 , 68	0	27	27
17 , 18	3	11	14	77 , 78	0	28	28
5 , 53	3	7	10	53 , 54	0	13	13

We see some motifs having relative large differences in prevalence between ASD group and TD group. These are the potential discriminating motifs. Essentially the fine scale pattern information that we try to extract comes from two distinct aspects of motif prevalence: 1) prevalence ratio as an odds; 2) number of missing motifs with respect to a restricted motif domain via a prevalence thresholding. We illustrate these two aspects of information content by taking motif-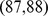 as an example. For the first aspect, the prevalence ratio of this motif in ASD group and TD group is 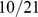. That is, a subject having such a motif is twice more likely to be coming from TD group than from ASD group. For the second aspect, let 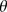 be a prevalence threshold. For instance 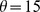, then restricted motif domain 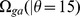 is a subset of 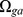 and is only consisting of motifs with prevalence being more than or equal to 15, so is 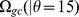 defined. Hence motif-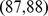 is not in 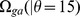, but in 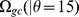. This membership difference becomes one piece of important pattern information since odds are only computed for motifs belonging to 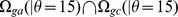. It is proved in the next section that these two aspects of extracted fine scale pattern information bear greater potential to discriminate ASD subjects from TD subjects than the coarse scale connectivity pattern information.

In Table 4, we provide the names of brain regions associated with a short list of potentially discriminating motifs, or functional brain connectivity via networking. This table clearly indicates the heterogeneity in motif prevalence.

**Table 4:** Potential motif's brain location.

Motif	ASD Prevalence	TD Prevalence	Name 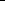 ROI1	Name 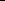 ROI2	Anatomical Region
87,88	10	21	Temporal 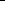 Inf 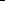 L	Temporal 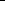 Inf 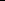 R	Temporal
63,79	13	21	Pallidum 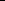 L	Putamen 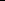 L	Subcortical
64,80	14	19	Pallidum 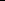 R	Putamen 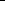 R	Subcortical
19,20	16	22	Cerebelum 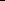 Crus1 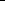 L	Cerebelum 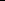 Crus1 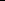 R	Cerebelum
51,52	16	21	Insula 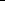 L	Insular 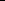 R	Insular
85,86	22	16	SupraMarginal 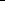 L	SupraMarginal 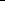 R	Parietal
74,76	21	19	PostCentral 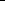 R	PreCentral 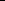 R	Central
73,75	18	15	PostCentral 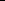 L	PreCentral 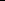 L	Central
91,92	19	20	Temporal 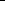 Pole 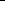 Mid 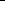 L	Temporal 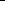 Pole 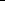 Mid 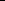 R	Limbic
40,44	19	15	Frontal 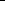 Mid 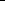 R	Frontal 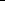 Sup 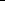 R	Frontal
37,41	17	13	Frontal 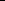 Mid 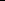 L	Frontal 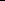 Sup 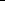 L	Frontal
48,82	18	12	Heschi 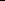 R	Rolandic 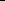 Oper 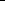 R	Temporal/Central
47,81	11	7	Heschi 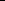 L	Rolandic 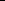 Oper 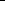 L	Temporal/Central

At the end of this section we briefly compare the two trees corresponding to the fine and coarse scales in Fig. 7 (a) and (b) with the HC-trees based on the “raw” correlation matrix in Fig. 7(c). This comparison intends to bring out why the “raw” HC tree could not retain the essential motif information. The key reason is its lacking of stability in motif configuration across different subjects. That is, a collection of branches resulted from any small threshold for pruning contains heterogeneous sizes. It is evident that it misses a significant proportion of motifs with left-and-right matching patterns, and at the same time contains large branches that are hardly observed again across different subjects. This unstable phenomenon can be partly attributed to the heterogeneity of hidden scales 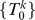, and partly to its employed construction procedure. Again we emphasize that a relational data matrix always needs scale-standardizing procedure in order to reveal embedded pattern information.

### Application to classification of ASD

We are interested in predicting the probability of someone belonging to ASD group based on the fine and coarse scale pattern information extracted from the previous sections. We construct a supervised learning algorithm based on the leave-one-out cross-validation procedure [5], [29] in this section. We first develop the logistic regression set-up for accommodating the two aspects of fine scale pattern information with a range of a prevalence threshold 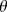 between 2 and 22. The extracted coarse scale pattern information would be accommodated into the logistic regression as covariates later.

#### Feature extraction

Consider restricted group-trial specific motif domains by leaving out one subject. Firstly, in the green trials and within the ASD group, a chosen subject 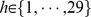, is to be left out from the ASD group of 29 subjects. The remaining 28 individuals' motif configurations under the threshold 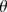 are assembled into a restricted motif domain 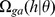 with motif prevalence function 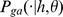. Secondly, within the TD group, we have 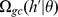 and 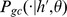 with 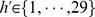 as the subject left out.

For the odds aspect of fine scale pattern information, we compute the ASD vs. TD group-membership odds for subject 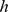 in ASD group on green trials based on its motif configuration 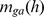. That is , we compute the odds for subsets of motifs 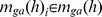 contained in both 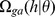 and 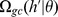 based on prevalence function 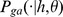 and 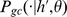:
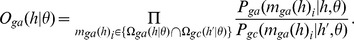



Similarly, for subject 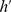 in TD group on green trials, we have the ASD vs. TD odds for the subsets of motifs 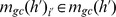 contained in both 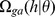 and 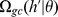 based on prevalence function 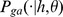 and 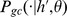:
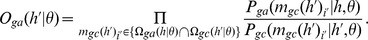



For the aspect of number of motifs missing from various restricted motif domains, we count two membership numbers for each individual motif configuration. For 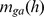, we count how many its members being missing in 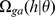 and 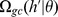, so is for 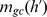. The two membership numbers are calculated in the following:
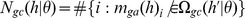


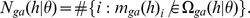



We calculate similar information 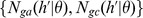 for subject 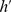 in TD group on green trials. Similarly we extract corresponding information for subject 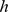 in ASD group and subject 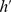 in TD group on red trials:
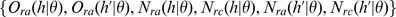
In summary we extract six-dimensional fine scale pattern information as
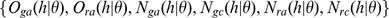
for subject 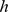 in ASD group, and
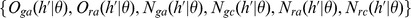
for subject 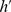 in TD group.

#### Logistic regression leave-one-out cross-validation

We then perform the following logistic regression leave-one-out cross-validation procedure. Response variable group membership 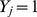 for ASD subjects 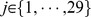 and 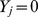 for TD subjects 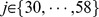. Four predictor variables are defined as transformation of the six-dimensional fine scale pattern information: 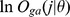, 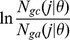, 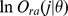, 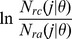, 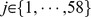.

Let 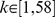 be the subject being left out in the leave-one-out cross-validation procedure, and the remaining 57 subjects as training data in the logistic regression. Denote the regression parameter estimate vector by 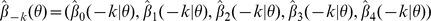 when leaving subject 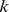 out. Then the predicted probability of 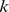 belonging to ASD group based on these leave-one-out logistic regression model coefficients will be calculated as
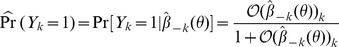
where
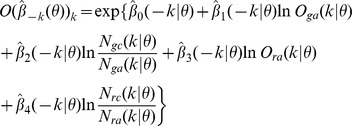



We then classify subject 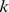 according to 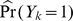: into ASD group if 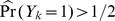, otherwise into TD group. By systematically varying subjects 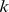, we are able to calculate the sensitivity 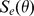 as the correct classification rate of ASD cases, and specificity 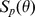 as the correct classification rate of TD cases.

## Results

In this section we report the results of our supervised learning rules based on coarse and fine scales pattern information. We first report 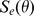 and 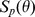 with 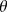 varying from 2 to 22 based on the fine scale patten information in Table 5. Based on the fine scale pattern information from the combination of green trials and red trials, 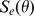 and 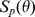 are rather high with 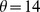. This empirical fact highlights that the counts of missing motifs are particularly informative. Its implication in functional brain connectivity is very clear: it is crucial to find one potential connection or motif being missing, and equally crucial to find an extra connection being present.

**Table 5:** Logistic regression leave-one-out cross-validation classification rate.

classification	green		green	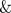 red	red	
prevalence 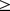	Se	Sp	Se	Sp	Se	Sp
2	10/29	10/29	12/29	14/29	13/29	14/29
3	14/29	13/29	16/29	14/29	19/29	16/29
4	13/29	14/29	19/29	19/29	21/29	22/29
5	16/29	16/29	15/29	16/29	15/29	15/29
6	13/29	15/29	18/29	18/29	19/29	17/29
7	16/29	17/29	15/29	20/29	15/29	14/29
8	17/29	13/29	17/29	19/29	20/29	18/29
9	17/29	17/29	17/29	16/29	19/29	17/29
10	16/29	19/29	18/29	18/29	19/29	20/29
11	19/29	18/29	21/29	23/29	19/29	19/29
12	16/29	17/29	19/29	18/29	16/29	16/29
13	17/29	15/29	17/29	20/29	14/29	17/29
**14**	22/29	19/29	**21/29**	**24/29**	19/29	20/29
15	18/29	20/29	16/29	18/29	16/29	16/29
16	17/29	20/29	20/29	20/29	18/29	16/29
17	22/29	21/29	21/29	21/29	16/29	15/29
18	17/29	19/29	18/29	20/29	13/29	16/29
19	22/29	17/29	19/29	19/29	20/29	18/29
20	16/29	17/29	12/29	15/29	7/29	6/29
21	20/29	22/29	19/29	25/29	20/29	17/29
22	15/29	16/29	14/29	14/29	15/29	17/29

Table 5 also reveals that incorporating fine scale pattern information from red trials only incrementally increases 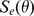
s and 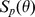
s. This empirical fact might not be surprising because the correlation matrices derived from red trials are less stable due to fewer trials comparing with the green ones.

Next we report the performance of all our supervised learning rules, which are likewise developed based on pattern information extracted at different levels, in Table 6. The fine scale pattern information is based on green and red trials with prevalence threshold at 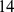. The third and fourth learning algorithms are results of model selection among the 10 extracted coarse scale information given in Table 1.

**Table 6:** Performance of four supervised learning rules.

Supervised learning rules	Sensitivity	Specificity
VS-coarse-1	18/29	19/29
VS-Fine	21/29	24/29
VS-Fine 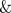 VS-coarse-2	24/29	24/29
VS-Fine 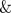 VS-coarse-3	24/29	24/29

**VS-Fine** Fine scale variable set: 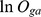, 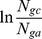, 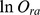, 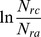 at prevalence threshold 14.

**VS-coarse-1** Coarse scale connectivity variable set 1: Within Parietal; Between Cerebelum and Central; Between Temporal and Limbic; Within Insular; Between Subcortical and Occipital; Between Limbic and Parietal; Between Limbic and Occipital; Between Subcortical and Frontal; Between Parietal and Occipital; Within Occipital.

**VS-coarse-2** Coarse-scale connectivity variable set 2: Within Insular; Between Subcortical and Occipital; Between Subcortical and Frontal; Within Occipital.

**VS-coarse-3** Coarse-scale connectivity variable set 3: Between Limbic and Parietal; Between Subcortical and Frontal; Between Parietal and Occipital; Within Occipital.

Based on the results in Table 6, we conclude that most of the predictive power of the logistic regression model resides at the level of the fine scale pattern information, suggesting that cellular versus systems level disturbances are more prominent in individuals with ASD.

## Discussion

In this study we demonstrate how to apply the data cloud geometry algorithm on relational connectivity data to construct the multiscale connectivity clustering geometry. We extract potential motifs as functional brain connectivity patterns on the fine scale level and potential regional abnormality of connectivity on the coarse level. The two different aspects of connectivity patterns are brought together for constructing a reasonably effective classification for ASD. One essential message derived from this study is the following: most relational connectivity data in a form of correlation matrix of ROIs are typically embedded with multi-scale structure. Different scales give rise to different information contents.

Our computational data cloud geometry based connectivity clustering geometry is potentially useful in neurosciences in general. More research efforts are needed in order to make our 3-D functional brain connectivity network (as “pseudo-neuroanatomy” together with the computed collection of motifs and their prevalence information) accessible for clinicians to “visualize” potential abnormal connectivity patterns among ROIs. Our computational supervised learning approach proposed in this paper has successfully addressed more general questions of how to detect functional brain connectivity patterns for classifying and diagnosing ASD. Though our computational and methodological developments are exclusively illustrated via ASD, they are equally applicable for other brain disorders.

Our computed fine scale information, including identifying a collection of motifs and their prevalence, significantly brings out the potential for discriminating abnormal and pathological ASD subjects. Similarly in [5], fine scale information, which is the exclusive presence of pairwise connectivity among 7266 ROIs, is also extracted. The discriminating potential is also demonstrated.

The coarse scale information via the high accordance between our connectivity clustering geometry and anatomical brain region is a more systematic alternative to approaches used in [25] and [30]. Though confirmations of status of short- and long-range vs. under- or over-connectivity across 10 brain regions are beyond the scope of this paper, our computed coarse scale information is shown to bear potential information for differentiating ASD and TD. In contrast the approaches used in [25] compared the ASD group's rates activation with that of control group among all anatomical regions. While the approaches used in [30] summarized structural MRI information into five dimensions and applied supported vector machine learning algorithm to derive the diagnostic test for ASD. Besides, the regions that discriminate autistic and non-autistic participants only partially overlap with regions identified by existing research [31].

We successfully develop a supervised learning rule, or so-called classification or diagnostic test for ASD, by combining the fine and coarse scales information. This methodological development could be applied to other brain disorders based on fMRI data. As for the effectiveness issue, we attempt to perform extensively tests in various simulation settings (see supplementary file S2). It is worthy noting that, though our simulated relational data sets are generated via a design mimicking intra- and inter-regional functional connectivity with respect to brain anatomical structure, our supervised learning rules always provide perfect classification on a wide spectrum of settings. This phenomenon clearly indicates that the subtlety and complexity embraced within real fMRI data is far beyond our modeling capabilities. In other words, how to realistically model and simulate brain's functional connectivity are very complex issues [32], [33].

Here again we reiterate that functional brain connectivity has the essential merit of elucidating how the anatomical architecture supports neurophysiological dynamics in brain [1]. This functional approach can also complement the approach of structural brain connectivity [34] in many areas of brain research, especially on linking the changes in connectivity patterns to biological and evolutionary phase shifts. Nevertheless the extent of merit and success via functional connectivity could well depend on how the connectivity is constructed and how patterns are extracted [33]. Thresholding based connectivity together with social network measurements are likely to have rather limited effectiveness.

Finally whether our results are clinically coherent with the under-connectivity hypothesis of autism being put forth in [4] needs further investigations. The hypothesis posits that autism is marked by under-functioning high-level neural connections and synchronization, along with an excess of low-level processes. Evidence for this theory has been found in functional neuroimaging studies on autistic individuals and by a brain wave study that suggested that adults with autism spectrum disorders (ASD) have local over-connectivity in the cortex and weak functional connections between the frontal lobe and the rest of the cortex. If such coherence is confirmed, our learning rule should accommodate this prior knowledge to be more effective.

## Supporting Information

Text S1 **The file contains the table of ROI names and coordinates.**
(PDF)

Text S2 **The file provides information related to simulation studies.**
(PDF)

Text S3 **The file contains one figure with fifty eight sub figures.** The figures display the hierarchical clustering tree of 106 ROIs for each subject in ASD group and TD group during green trials based on ensemble matrix obtained at T = 0.001.
(PDF)
