# Supplementary material for: Extracting Multiscale Pattern Information of fMRI Based Functional Brain Connectivity with Application on Classification of Autism Spectrum Disorders
Source: PLoS One. 2012 Oct 8;7(10):e45502. doi: 10.1371/journal.pone.0045502 (PMC3466274; doi:10.1371/journal.pone.0045502)
Supplement: Text S1 — The file contains the table of ROI names and coordinates. (PDF) [file pone.0045502.s001.pdf]

Supplementary Material S1 for

Extracting multiscale pattern information of fMRI based functional brain connectivity with application on classification of autism spectrum disorders

Hui Wang[1], Chen Chen[1], Hsieh Fushing[1,*]
1 Department of Statistics, University of California, Davis, One Shields Ave., Davis, CA, USA 95616
∗ **E-mail: fushing@wald.ucdavis.edu**

This file contains only a table.

Table 1. ROI names and coordinates

ID	Name	x	y	z	ID	Name	x	y	z
1	Amygdala_L	-23.5	-1.95	-18.5	54	Lingual_R	16.1	-68.1	-5.22
2	Amygdala_R	27.1	-0.573	-18.8	55	Occipital_Inf_L	-36.5	-79.6	-9.16
3	Angular_L	-44.4	-62.1	34.3	56	Occipital_Inf_R	37.9	-83.2	-8.99
4	Angular_R	45.2	-61.2	37.3	57	Occipital_Mid_L	-32.6	-82.0	14.8
5	Calcarine_L	-7.48	-79.8	5.10	58	Occipital_Mid_R	37.1	-81.0	18.1
6	Calcarine_R	15.7	-74.4	8.02	59	Occipital_Sup_L	-16.8	-85.6	26.9
7	Caudate_L	-11.8	9.72	8.07	60	Occipital_Sup_R	24.0	-82.2	29.3
8	Caudate_R	14.5	10.8	8.15	61	Olfactory_L	-8.26	13.9	-12.7
9	Cerebelum_10_L	-8.12	-38.4	-19.8	62	Olfactory_R	10.1	14.7	-12.6
10	Cerebelum_10_R	13.1	-35.7	-20.6	63	Pallidum_L	-18.1	-1.36	-10.4
11	Cerebelum_3_L	-14.3	-44.7	-18.3	64	Pallidum_R	20.9	-1.06	-1.09
12	Cerebelum_3_R	17.9	-44.2	-19.4	65	ParaHippocampal_L	-21.5	-17.3	-21.9
13	Cerebelum_4_5_L	-22.5	-60.3	-23.5	66	ParaHippocampal_R	25.1	-16.3	-21.7
14	Cerebelum_4_5_R	25.4	-59.5	-24.9	67	Paracentral_Lobule_L	-7.97	-26.7	68.7
15	Cerebelum_6_L	-31.6	-61.0	-46.8	68	Paracentral_Lobule_R	7.13	-32.9	66.8
16	Cerebelum_6_R	33.9	-64.4	-49.8	69	Parietal_Inf_L	-43.1	-47.0	45.4
17	Cerebelum_9_L	-21.8	-35.0	-43.1	70	Parietal_Inf_R	46.3	-47.6	48.2
18	Cerebelum_9_R	26.8	-35.0	-42.6	71	Parietal_Sup_L	-23.7	-60.8	57.7
19	Cerebelum_Crus1_L	-35.4	-67.9	-30.3	72	Parietal_Sup_R	25.8	-60.4	60.7
20	Cerebelum_Crus1_R	38.2	-68.4	-30.8	73	Postcentral_L	-42.9	-23.8	47.5
21	Cingulum_Ant_L	-4.36	34.2	12.5	74	Postcentral_R	41.2	-26.8	51.3
22	Cingulum_Ant_R	8.12	35.7	14.4	75	Precentral_L	-38.9	-6.96	49.6
23	Cingulum_Mid_L	-5.88	-16.1	40.2	76	Precentral_R	41.1	-9.55	50.8
24	Cingulum_Mid_R	7.66	-10.2	38.4	77	Precuneus_L	-7.59	-57.3	46.6
25	Cingulum_Post_L	-5.21	-44.2	23.3	78	Precuneus_R	9.69	-57.3	42.4
26	Cingulum_Post_R	7.18	-43.1	20.5	79	Putamen_L	-24.2	2.60	1.07
27	Cuneus_L	-6.26	-81.4	25.8	80	Putamen_R	27.5	3.68	1.19
28	Cuneus_R	13.2	-80.6	26.9	81	Rolandic_Oper_L	-47.4	-9.78	12.6
29	Frontal_Inf_Oper_L	-48.8	11.5	17.8	82	Rolandic_Oper_R	52.4	-7.54	13.3
30	Frontal_Inf_Oper_R	49.9	13.7	20.2	83	Supp_Motor_Area_L	-5.69	3.55	60.1
31	Frontal_Inf_Orb_L	-36.2	29.5	-13.5	84	Supp_Motor_Area_R	8.25	-1.09	60.5
32	Frontal_Inf_Orb_R	40.9	31.0	-13.3	85	SupraMarginal_L	-56.1	-34.9	29.1
33	Frontal_Inf_Tri_L	-45.9	28.7	12.6	86	SupraMarginal_R	57.3	-32.8	33.1
34	Frontal_Inf_Tri_R	50.1	28.9	12.8	87	Temporal_Inf_L	-50.0	-29.3	-24.5
35	Frontal_Med_Orb_L	-5.44	52.5	-8.86	88	Temporal_Inf_R	53.4	-32.1	-23.7
36	Frontal_Med_Orb_R	7.83	50.4	-8.52	89	Temporal_Mid_L	-55.9	-35.0	-3.58
37	Frontal_Mid_L	-33.8	31.5	34.1	90	Temporal_Mid_R	57.2	-38.6	-2.78
38	Frontal_Mid_Orb_L	-30.9	49.1	-11.0	91	Temporal_Pole_Mid_L	-36.7	13.3	-35.4
39	Frontal_Mid_Orb_R	32.9	51.3	-12.1	92	Temporal_Pole_Mid_R	44.0	13.2	-33.5
40	Frontal_Mid_R	37.4	31.8	32.8	93	Temporal_Pole_Sup_L	-40.2	13.9	-21.4
41	Frontal_Sup_L	-18.8	33.5	41.0	94	Temporal_Pole_Sup_R	47.9	13.5	-18.2
42	Frontal_Sup_Medial_L	-5.17	47.9	29.6	95	Temporal_Sup_L	-53.4	-22.0	5.84
43	Frontal_Sup_Medial_R	8.75	49.5	28.9	96	Temporal_Sup_R	57.8	-23.0	5.42
44	Frontal_Sup_R	21.6	29.9	42.5	97	Thalamus_L	-11.2	-18.8	6.61
45	Fusiform_L	-31.4	-41.4	-21.6	98	Thalamus_R	12.7	-18.8	6.73
46	Fusiform_R	33.7	-40.2	-21.5	99	Vermis_10	1.14	-47.1	-32.9
47	Heschl_L	-42.3	-20.0	8.68	100	Vermis_1_2	1.51	-40.1	-21.4
48	Heschl_R	45.9	-18.1	9.04	101	Vermis_3	2.05	-41.3	-12.7
49	Hippocampus_L	-25.3	-22.0	-11.4	102	Vermis_4_5	1.83	-53.6	-7.40
50	Hippocampus_R	28.9	-21.0	-11.6	103	Vermis_6	1.77	-68.4	-16.4
51	Insula_L	-35.4	5.40	2.17	104	Vermis_7	1.87	-73.2	-26.4
52	Insula_R	38.7	5.02	0.814	105	Vermis_8	1.81	-65.7	-35.3
53	Lingual_L	-14.9	-68.9	-6.01	106	Vermis_9	1.61	-56.2	-36.3
