# Supplementary material for: Extracting Multiscale Pattern Information of fMRI Based Functional Brain Connectivity with Application on Classification of Autism Spectrum Disorders
Source: PLoS One. 2012 Oct 8;7(10):e45502. doi: 10.1371/journal.pone.0045502 (PMC3466274; doi:10.1371/journal.pone.0045502)
Supplement: Text S2 — The file provides information related to simulation studies. (PDF) [file pone.0045502.s002.pdf]

Supplementary Material S2 for

Extracting multiscale pattern information of fMRI based functional brain connectivity with application on classification of autism spectrum disorders

Hui Wang[1], Chen Chen[1], Hsieh Fushing[1,*]
1 Department of Statistics, University of California, Davis, One Shields Ave., Davis, CA, USA 95616
∗ E-mail: fushing@wald.ucdavis.edu

This file provides information related to simulation studies.

Simulation Setting

In this section two sets of simulated data are offered on which we display the effectiveness of the proposed method. To mimic the brain fMRI data, two groups, autistic and control are simulated with 29 subjects in each of them. A 106×106 matrix is simulated to represent the brain activity correlation of each subject.

Anatomically, the 106 regions of interest (ROI's) are be classified into 11 clusters based on their physical locations in the brain (In the simulation study, ROIs of vermis are grouped together as a separate cluster). Compared to a pair of two ROI's in different clusters, a pair of ROI's within the same cluster is more highly correlated . Within each of the clusters, usually the pair of the ROI's with same functions (one in the left hemisphere and the other one in the right) is even more highly correlated. To accurately include this characteristic in the simulated data, a multi-scaled correlation matrix for each subject is needed.

Table 1. ROIs contained in each cluster

Cluster	1	2	3	4	5	6
ROIs	$1-10$	$11-20$	$21-34$	$35-46$	$47-68$	$69-76$
Cluster	7	8	9	10	11	
ROIs	$77-84$	$85-86$	$87-92$	$93-100$	$101-106$	

Another important property to be included in the simulation is the difference between ASD group and TD group. In each group, the correlation matrices are not exactly the same, but slightly different from subject to subject. However, the correlation differences between the two clinic groups are assumed to be more dominant.

Here we offer the approach by which we generate the first set of correlation matrices:

Step - 1, Generate two 106×106 distance matrices. One of them represents the distance measure on the ROI's from the control group, and the other one for the autistic group. To reflect the multi-scale characteristic, the distance between two ROI's in distinct anatomical regions is assumed to be from a normal distribution $\mathcal{N}(5,1)$. In contrast, the distance between two ROI's within an anatomical region is to be from $\mathcal{N}(2,0.5)$ which is expected to be smaller. At the finest scale, the distance between a left-right ROI pair is expected to be even shorter, from distribution $\mathcal{N}(1,0.2)$.

To illustrate the difference between two clinical groups, we break the ROI connections at different probabilities. By breaking the connection between two ROI's which are in the same anatomical region, the distance of the connection is reset to be from $\mathcal{N}(5,1)$. The breaking probabilities of the two groups are listed in Table 2.

Table 2. Breaking probabilities of each region in the first set of data

Region	1	2	3	4	5	6	7	8	9	10	11
prob. (control)	0.3	0.3	0.2	0.3	0.4	0.4	0.3	0.3	0.3	0.2	0.3
prob. (autism)	0.3	0.3	0.3	0.4	0.2	0.3	0.2	0.3	0.3	0.4	0.3

All the L-R pairs are broken at probability 0.2. If broken the distance between the L-R pair is drawn from $\mathcal{N}(2,0.5)$.

Step - 2, Generate the correlation matrices They are generated by applying data cloud geometry. For each distance, 29 ensemble matrices are generated at temperatures which are uniformly chosen from 0.3 to 0.7. The elements in the ensemble matrices are between 0 and 1, representing the correlations. Different temperatures yield correlations at distinct scales which are observed from the real fMRI data.

The second set of data are generated in the same fashion except that the two distance matrices are closer, with more similar breaking probabilities between the controls and the autistics. The breaking probabilities

are given in Table 3.

Table 3. Breaking probabilities of each region in the second set of data

Region	1	2	3	4	5	6	7	8	9	10	11
prob. (control)	0.3	0.3	0.25	0.3	0.3	0.35	0.3	0.3	0.3	0.3	0.3
prob. (autism)	0.3	0.3	0.3	0.3	0.3	0.3	0.3	0.3	0.3	0.25	0.3

Classification Algorithm

1 Given a correlation matrix $\rho[i,j]_{\{1\leq i,j\leq 106\}}$, a similarity matrix is constructed as $\mathrm{S}[i,j] = |\rho[i,j]|^{1/T}$, here $T = 0.005$ for both of the two simulated data sets, which is a low scale (temperature) used to reveal the finest clustering structure.

2 Using the regulated random walk, we used the "data cloud geometry" algorithm in Fushing and Michael 2010 paper with the parameter visit=5, iterations=1000 to obtain the ensemble matrix $\mathrm{Ens}[i,j]_{\{1\leq i,j\leq 106\}}$, i.e. cluster-sharing probability matrix.

3 With $\mathrm{dist}[i,j]_{\{1\leq i,j\leq 106\}} = 1 - \mathrm{Ens}[i,j]_{\{1\leq i,j\leq 106\}}$ as the distance matrix, we draw the hierarchical clustering tree.

4 We cut the trees at height=0.15 to obtain multiple branches (motifs) for each subject.

5 We count the prevalence of each motif for the autism group and control group. Then for subject i in autism group, we compare its motif collection to the collections of motifs of the rest autism subjects, and the collections of motifs of the corresponding rest control subjects.

6 If a motif exists in both autism and control collection, we calculate the ratio of the prevalence of it in autism and control collection, and we multiply the ratios together for all of the motifs of this subject.

7 If a motif doesn't exist in autism group, we record it as 1 missing link, then we add all the missing links together to obtain a count named number of new motifs to autism group; In a similar manner, we obtain the number of new motifs to control group; And the number of new motifs to both autism group and control group.

8 We use the ratio in item 6 and ratio of number of new motifs to two groups as the predictors in a leave-one-out cross-validation logistic regression.

9 Sensitivity and specificity are then calculated.

Classification

We extract the motifs as illustrated in the procedures above; Sensitivity and specificity are calculated based on leave-one-out cross validation logistic regression, and we obtain 100% sensitivity and specificity for all these simulated data sets, which reveals that multi-scale geometry works well in detecting the systematic difference between groups.

Clustering as a backup

We define the distance between two hierarchical clustering trees of two subjects A and B (see procedure above, item 3) as

$$d_{AB} = 1 - \frac{\#\{\text{matched motifs in (A,B)}\}}{\max\{\#\text{motifs in } A, \#\text{motifs in } B\}}.$$

Then we obtain the distance matrix of 58 subjects in the simulated data. The hierarchical clustering tree is shown below:

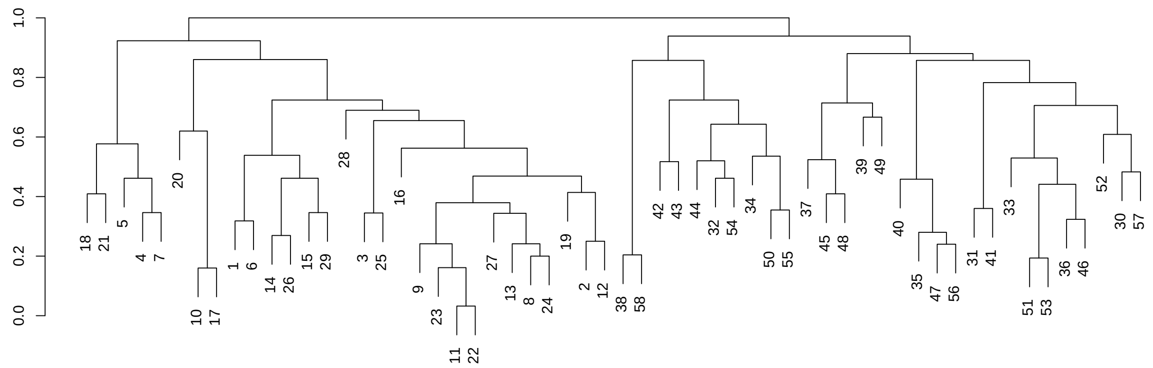

Figure 1. Hierarchical Clustering Tree of 58 Subjects Based on Common Motifs for Simulated Dataset 1.

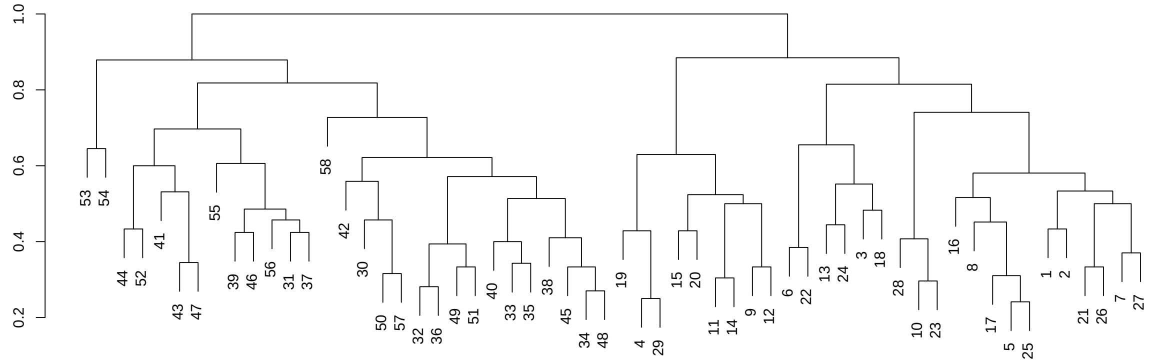

Figure 2. Hierarchical Clustering Tree of 58 Subjects Based on Common Motifs for Simulated Dataset 2.

From the tree, we notice all the 29 subjects in the first group are grouped together and the left 29 subjects are grouped together, which is consistent with the classification results in which we have 100% sensitivity and specificity respectively.
