# Supplementary material for: Extracting Multiscale Pattern Information of fMRI Based Functional Brain Connectivity with Application on Classification of Autism Spectrum Disorders
Source: PLoS One. 2012 Oct 8;7(10):e45502. doi: 10.1371/journal.pone.0045502 (PMC3466274; doi:10.1371/journal.pone.0045502)
Supplement: Text S3 — The file contains one figure with fifty eight sub figures. The figures display the hierarchical clustering tree of 106 ROIs for each subject in ASD group and TD group during green trials based on ensemble matrix obtained at T = 0.001. (PDF) [file pone.0045502.s003.pdf]

Supplementary Material S3 for

Extracting multiscale pattern information of fMRI based functional brain connectivity with application on classification of autism spectrum disorders

Hui Wang[1], Chen Chen[1], Hsieh Fushing[1,*]

1 Department of Statistics, University of California, Davis, One Shields Ave., Davis, CA, USA 95616

$*$ **E-mail: fushing@wald.ucdavis.edu**

This file contains one figure with fifty-eight sub figures.

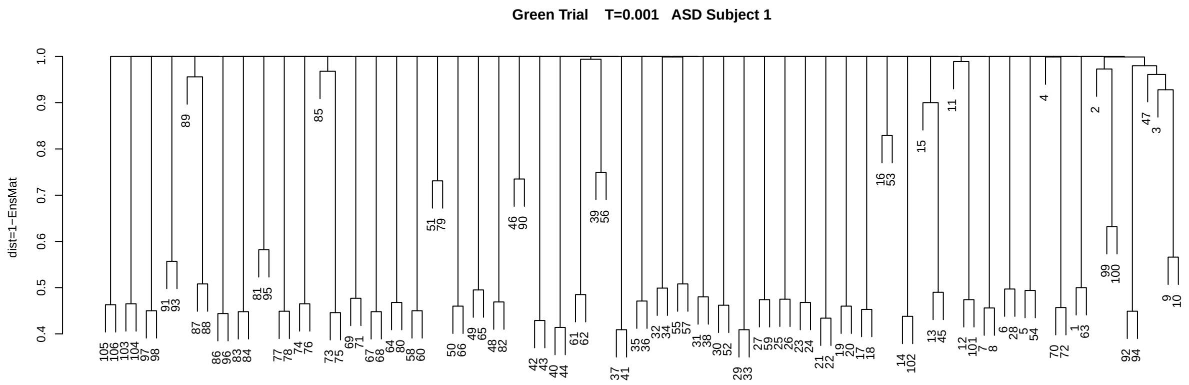
Green Trial T=0.001 ASD Subject 1
dist=1−EnsMat

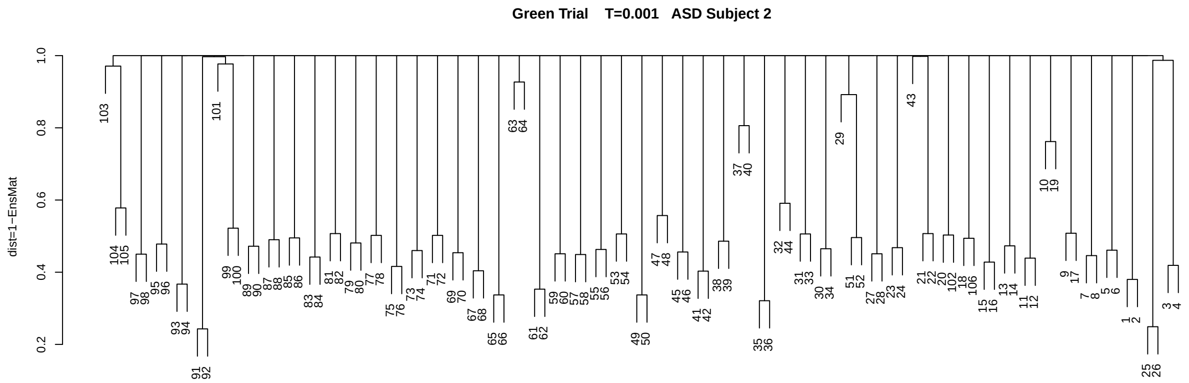
Green Trial T=0.001 ASD Subject 2
dist=1−EnsMat

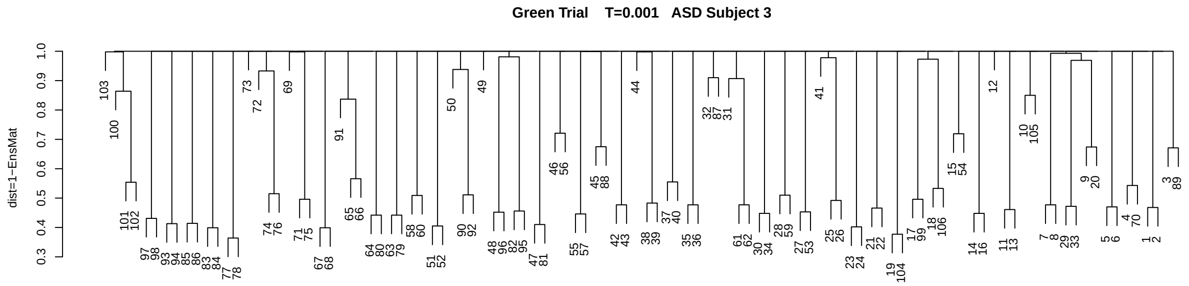
Green Trial T=0.001 ASD Subject 3
dist=1−EnsMat

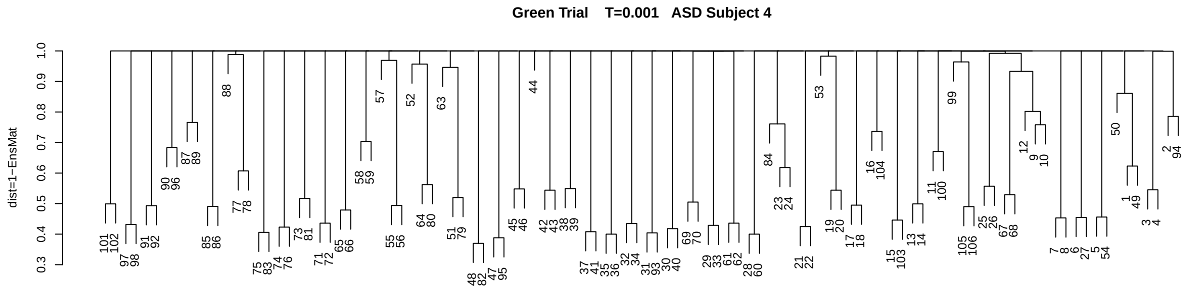
Green Trial T=0.001 ASD Subject 4
dist=1−EnsMat

Green Trial T=0.001 ASD Subject 5

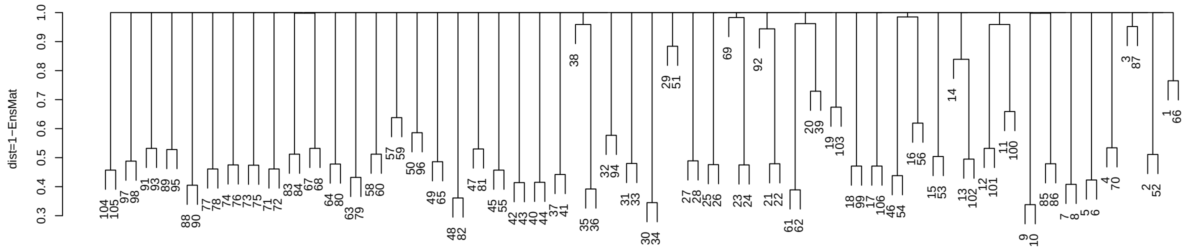

Green Trial T=0.001 ASD Subject 6

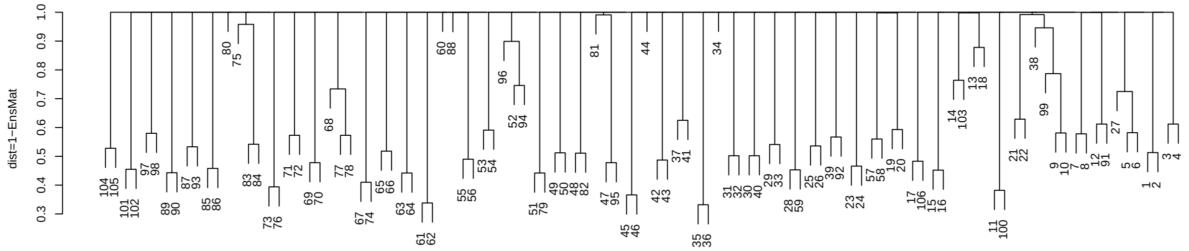

Green Trial T=0.001 ASD Subject 7

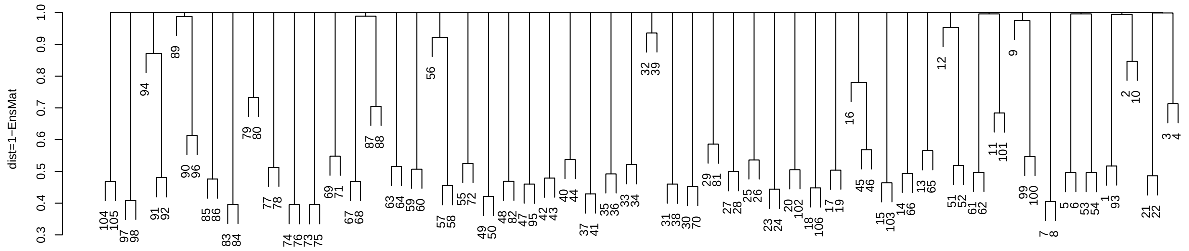

Green Trial T=0.001 ASD Subject 8

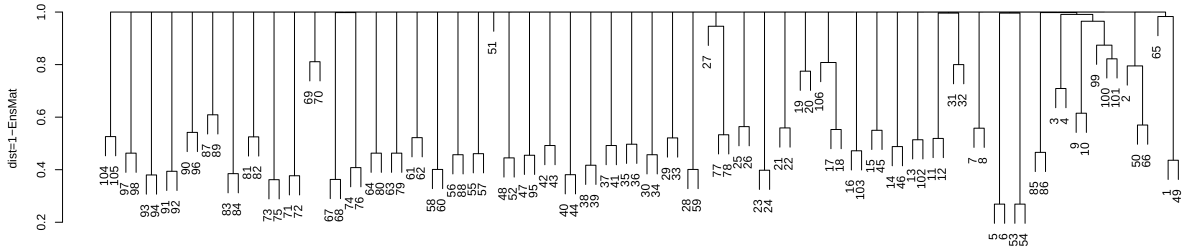

Green Trial T=0.001 ASD Subject 9

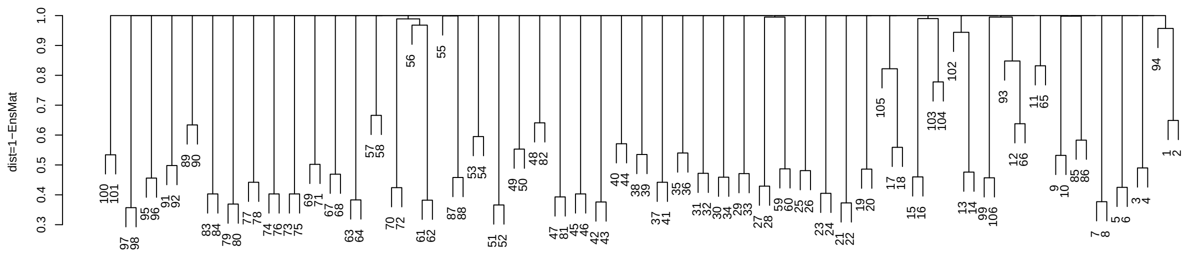

Green Trial T=0.001 ASD Subject 10

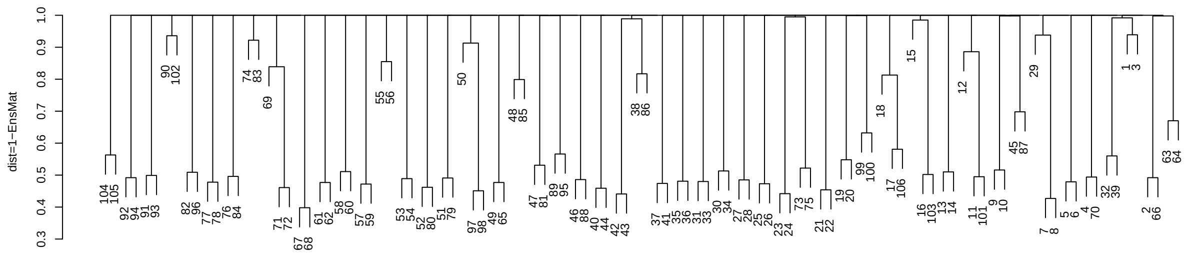

Green Trial T=0.001 ASD Subject 11

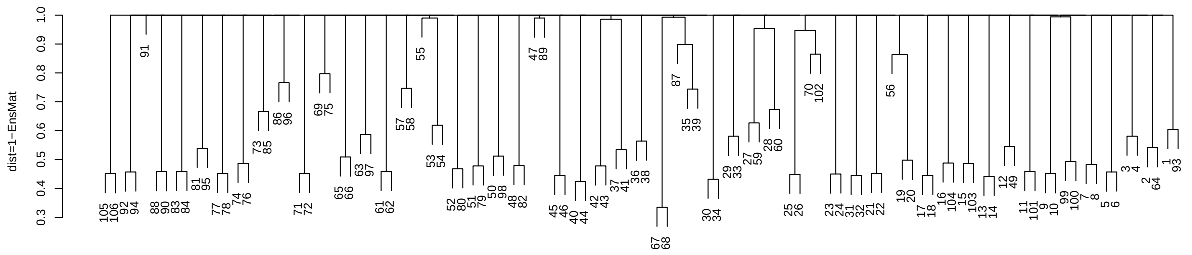

Green Trial T=0.001 ASD Subject 12

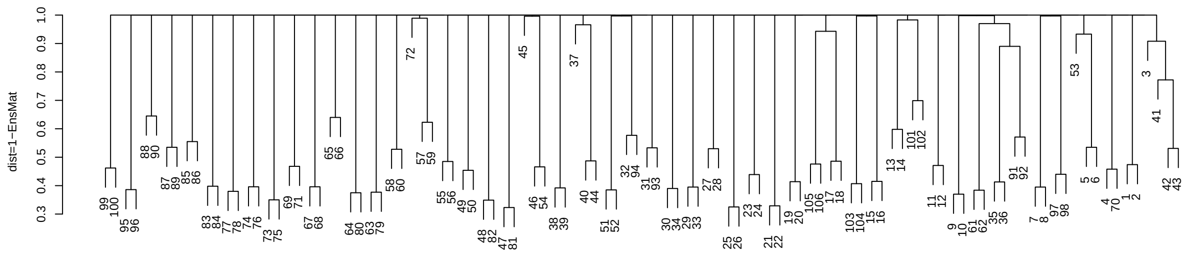

Green Trial T=0.001 ASD Subject 13

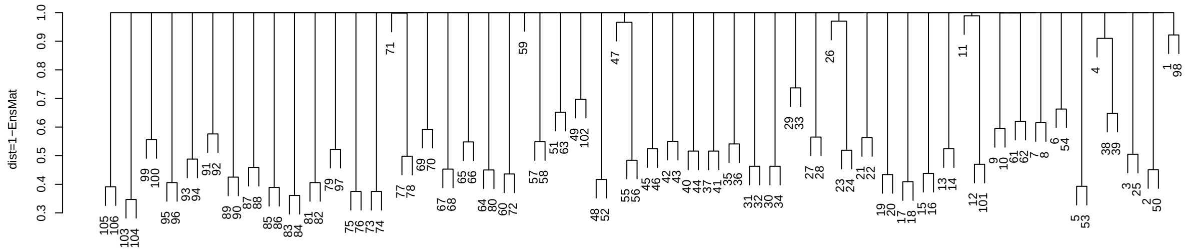

Green Trial T=0.001 ASD Subject 14

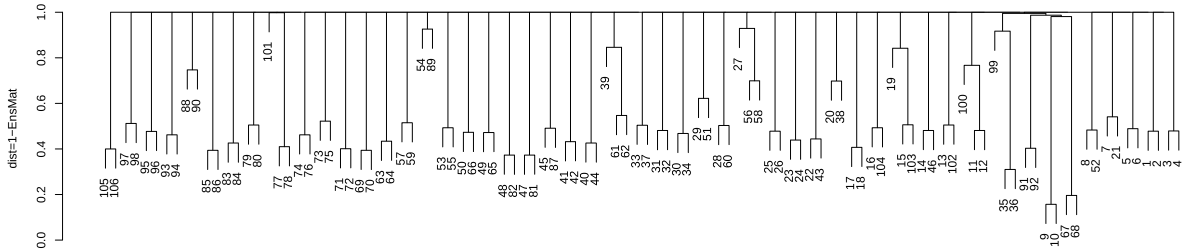

Green Trial T=0.001 ASD Subject 15

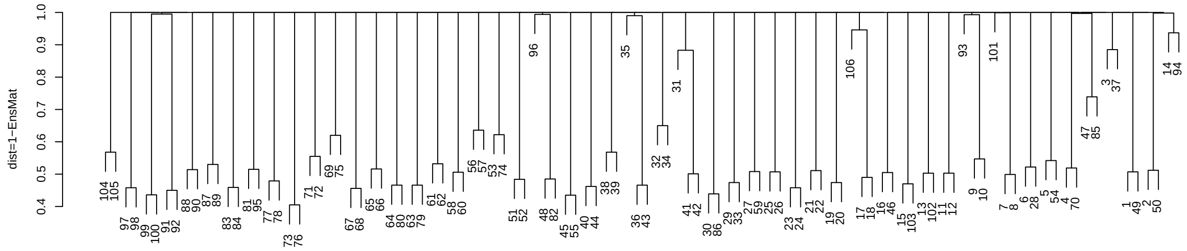

Green Trial T=0.001 ASD Subject 16

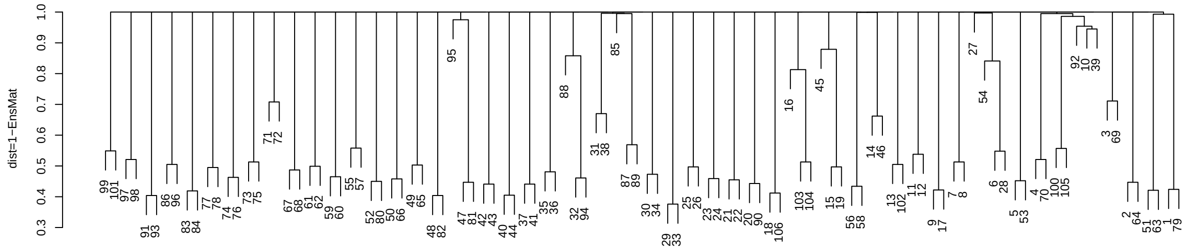

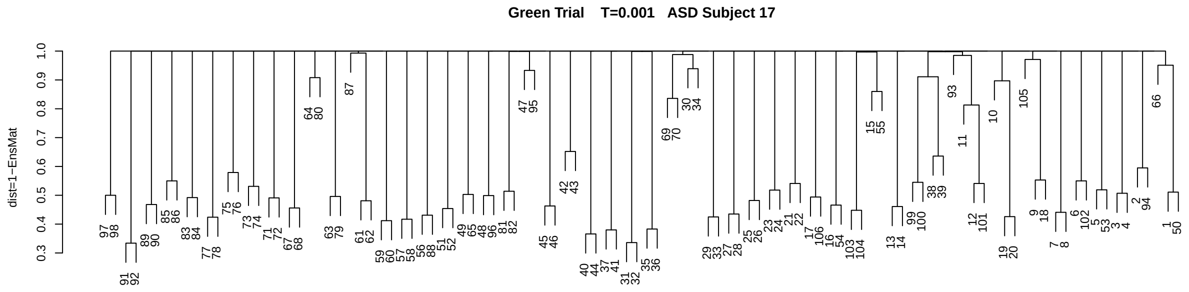
Green Trial T=0.001 ASD Subject 17
dist=1−EnsMat

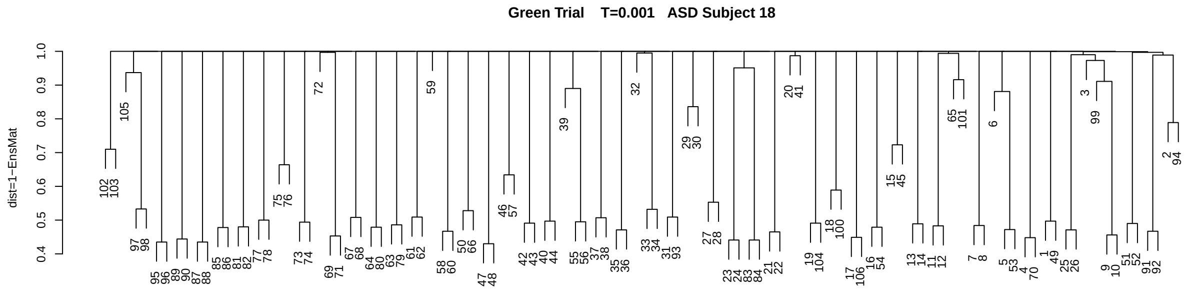
Green Trial T=0.001 ASD Subject 18
dist=1−EnsMat

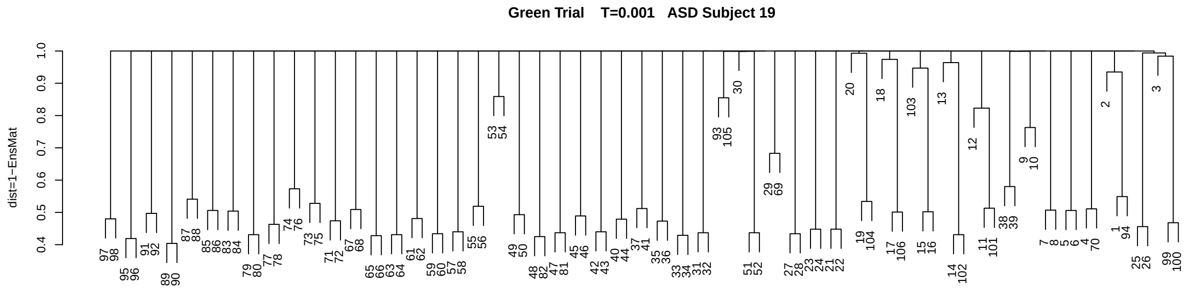
Green Trial T=0.001 ASD Subject 19
dist=1−EnsMat

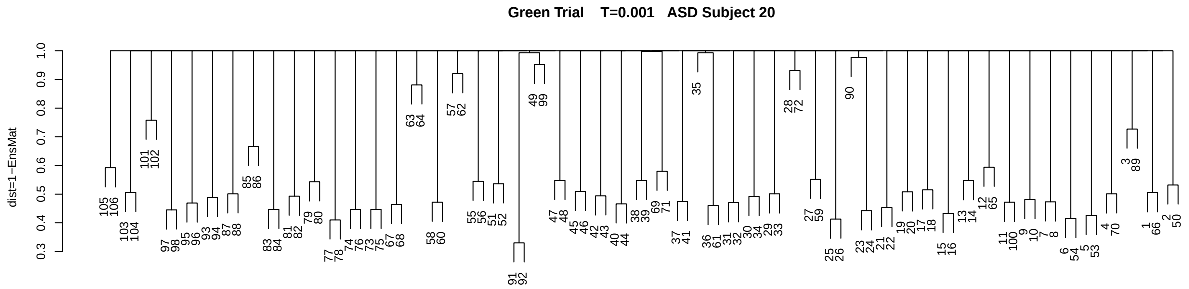
Green Trial T=0.001 ASD Subject 20
dist=1−EnsMat

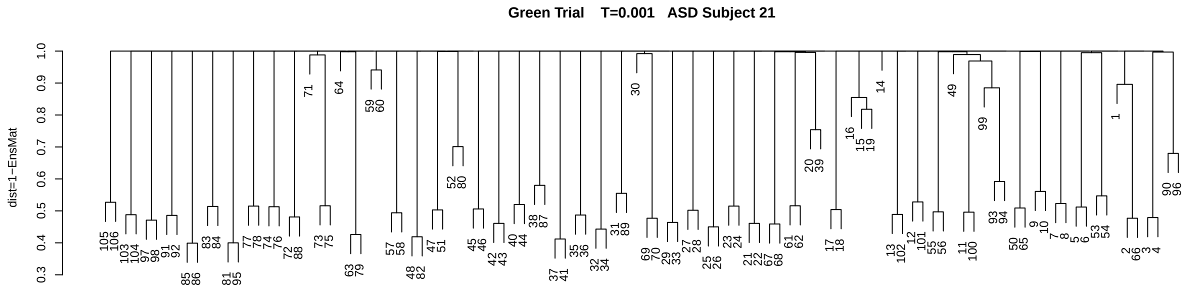
Green Trial T=0.001 ASD Subject 21
dist=1−EnsMat

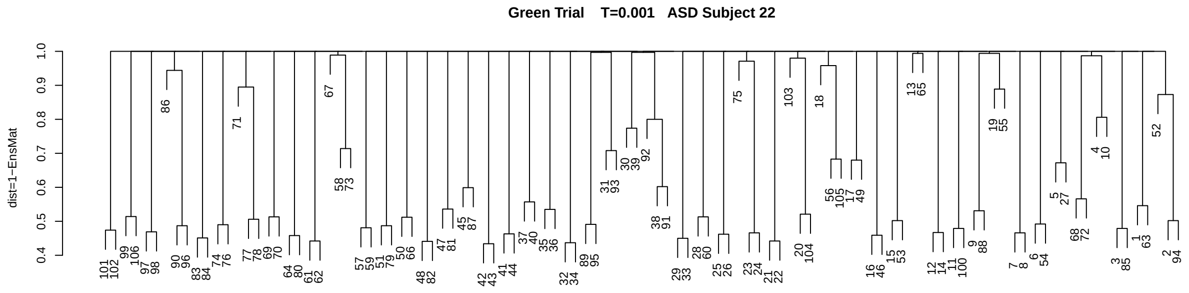
Green Trial T=0.001 ASD Subject 22
dist=1−EnsMat

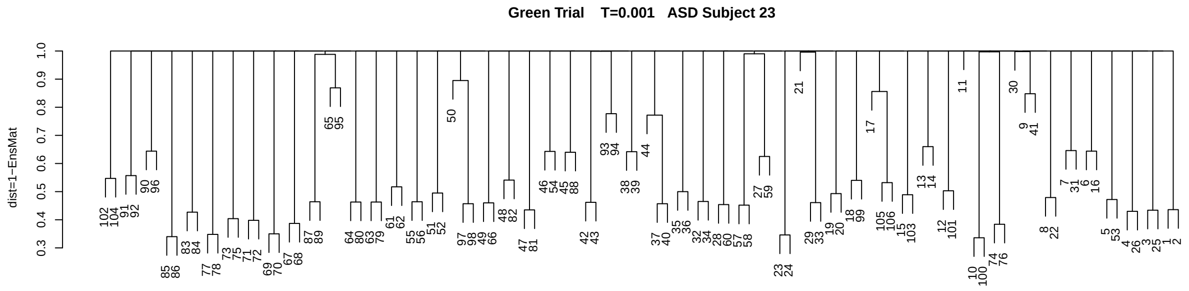
Green Trial T=0.001 ASD Subject 23
dist=1−EnsMat

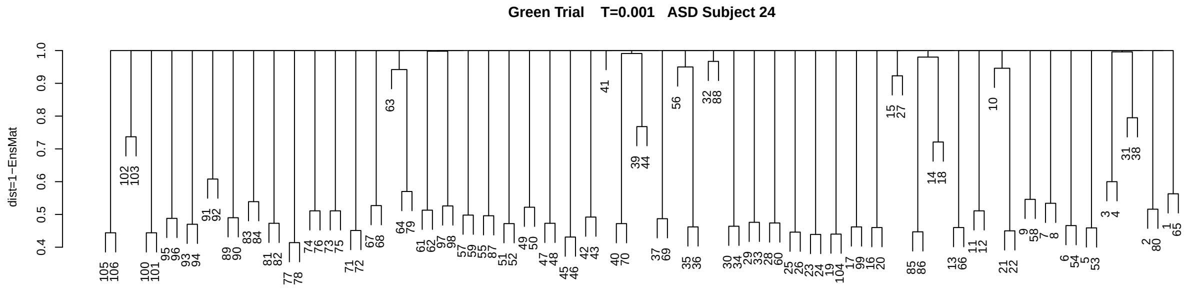
Green Trial T=0.001 ASD Subject 24
dist=1−EnsMat

Green Trial T=0.001 ASD Subject 25

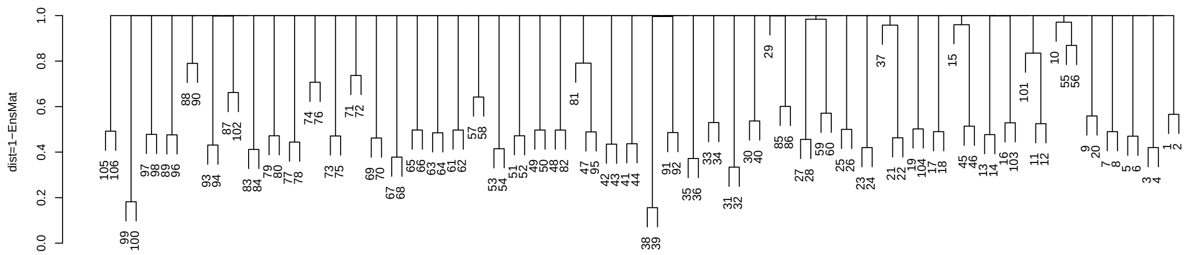

Green Trial T=0.001 ASD Subject 26

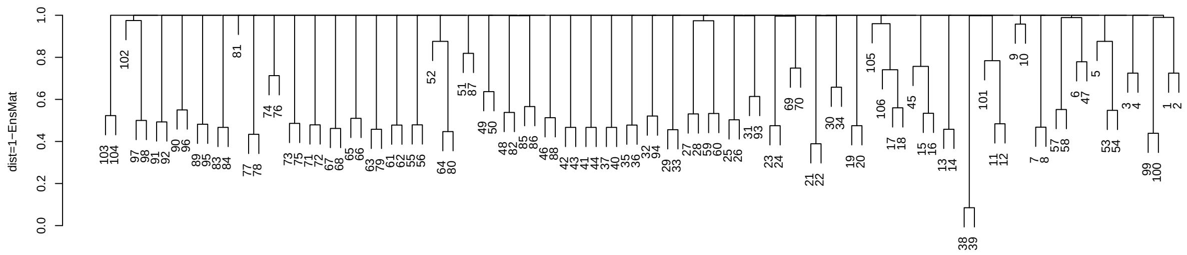

Green Trial T=0.001 ASD Subject 27

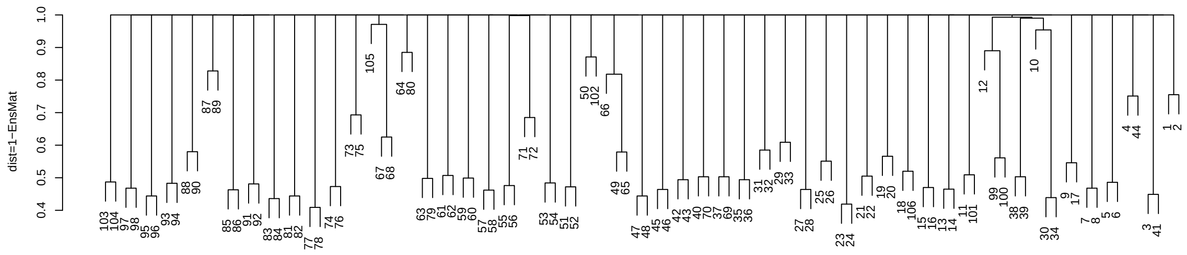

Green Trial T=0.001 ASD Subject 28

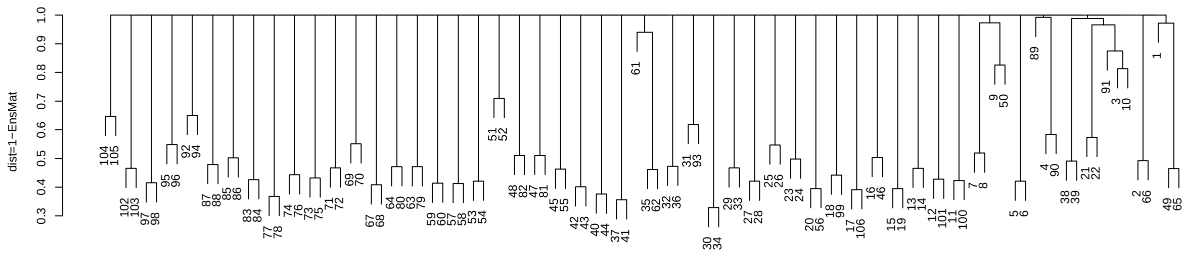

Green Trial T=0.001 ASD Subject 29

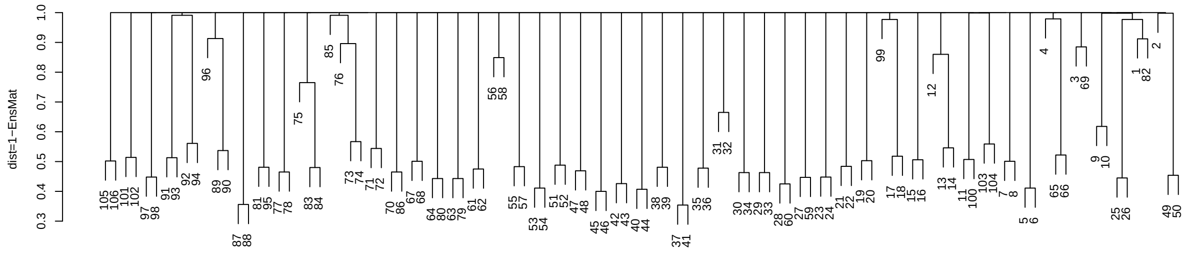

Green Trial T=0.001 Control Subject 1

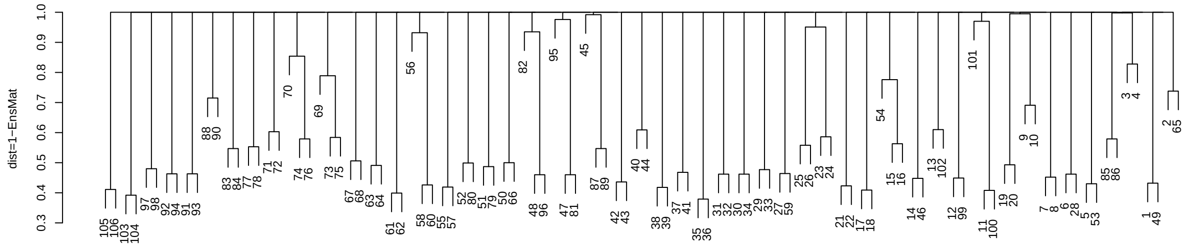

Green Trial T=0.001 Control Subject 2

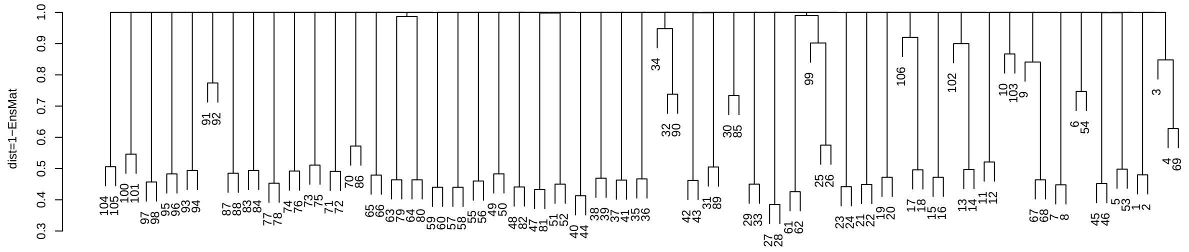

Green Trial T=0.001 Control Subject 3

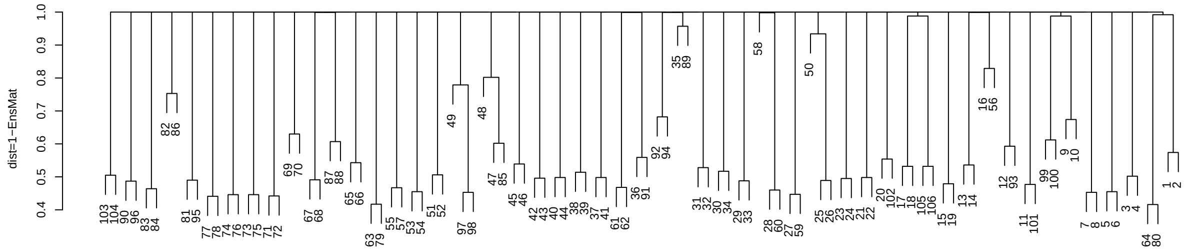

Green Trial   T=0.001   Control Subject 4

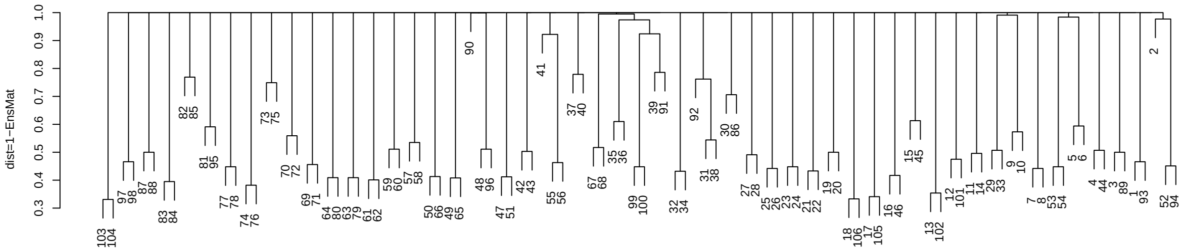

Green Trial   T=0.001   Control Subject 5

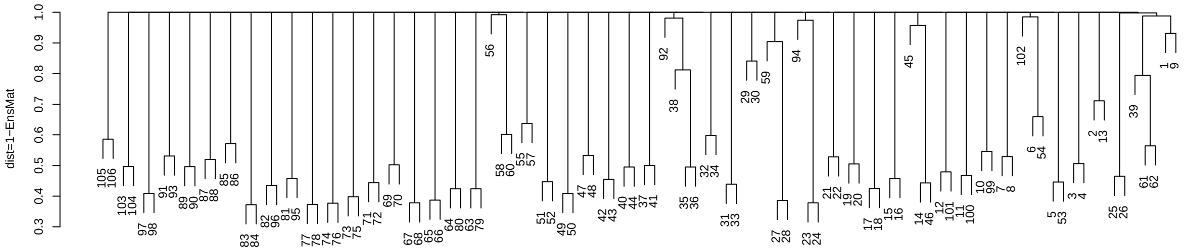

Green Trial   T=0.001   Control Subject 6

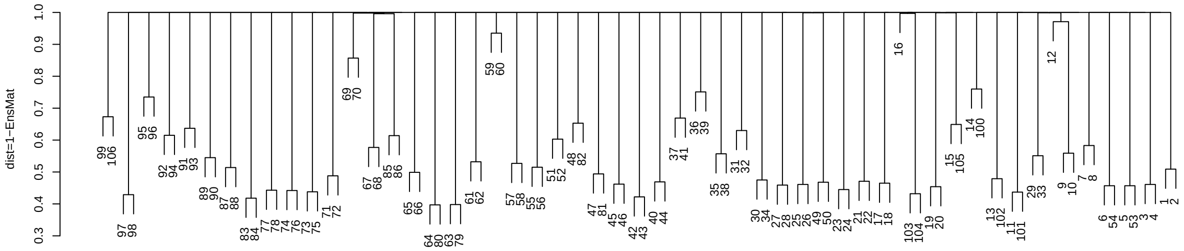

Green Trial   T=0.001   Control Subject 7

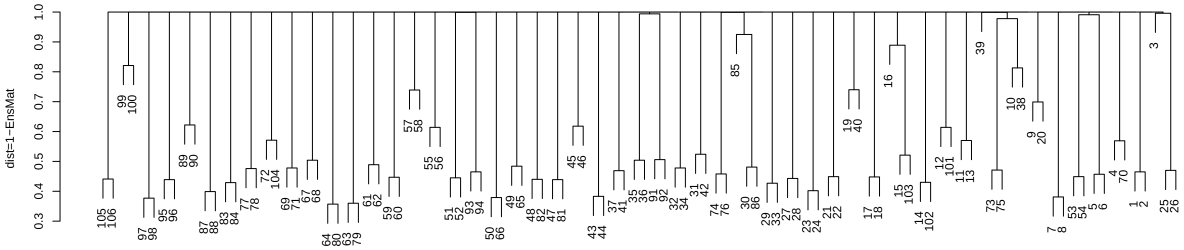

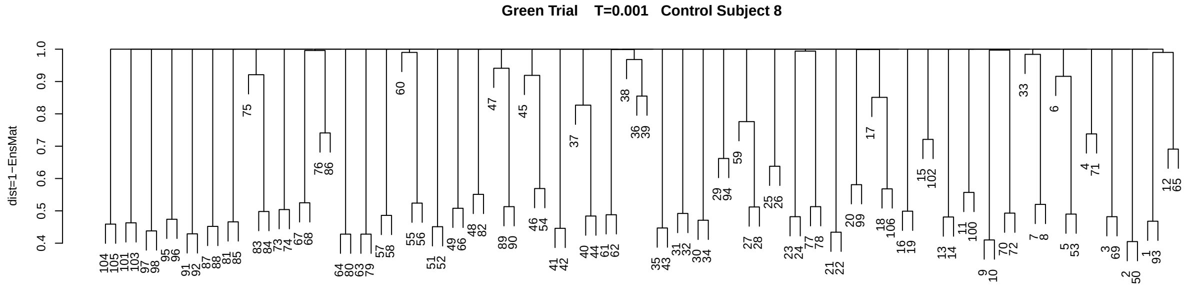
Green Trial T=0.001 Control Subject 8
dist=1−EnsMat

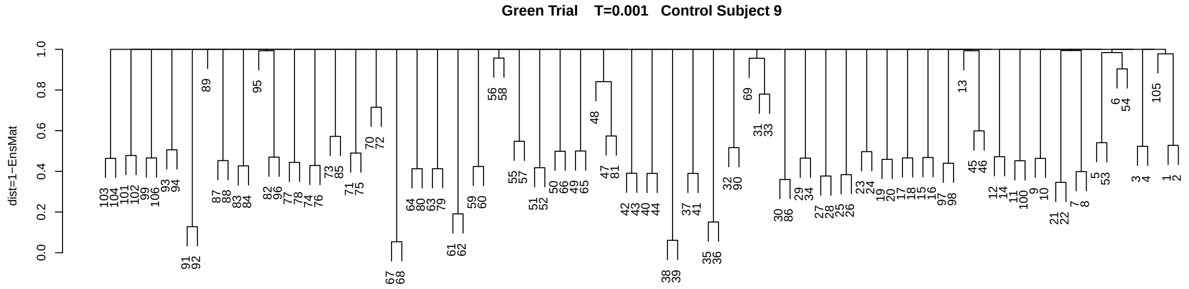
Green Trial T=0.001 Control Subject 9
dist=1−EnsMat

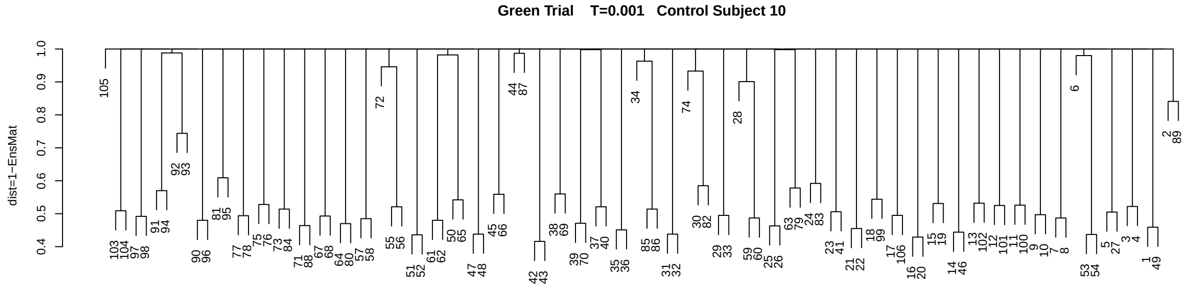
Green Trial T=0.001 Control Subject 10
dist=1−EnsMat

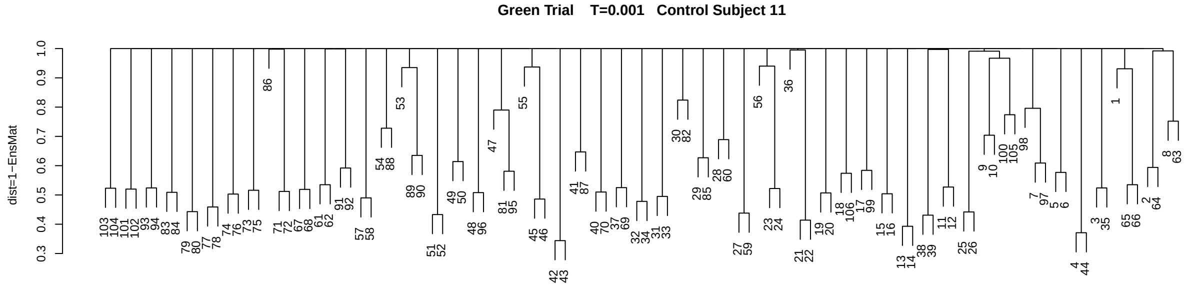
Green Trial T=0.001 Control Subject 11
dist=1−EnsMat

Green Trial T=0.001 Control Subject 12

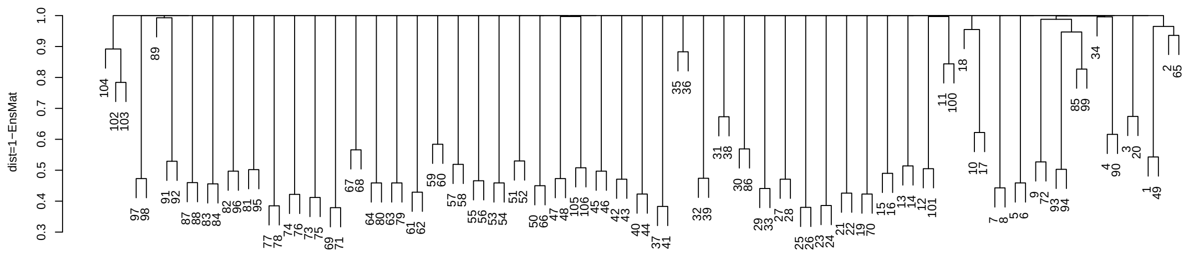

Green Trial T=0.001 Control Subject 13

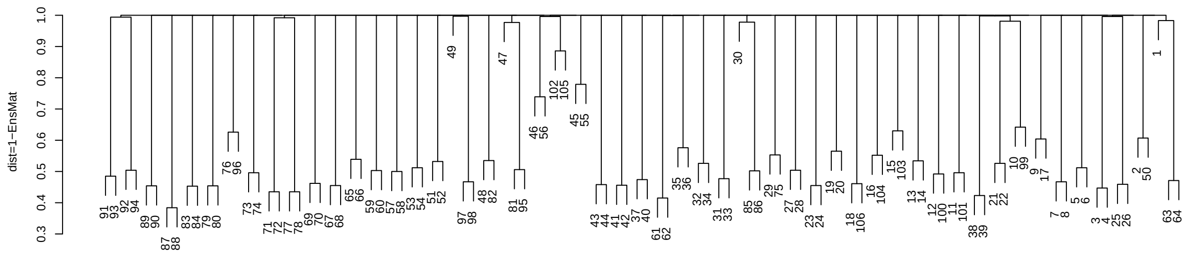

Green Trial T=0.001 Control Subject 14

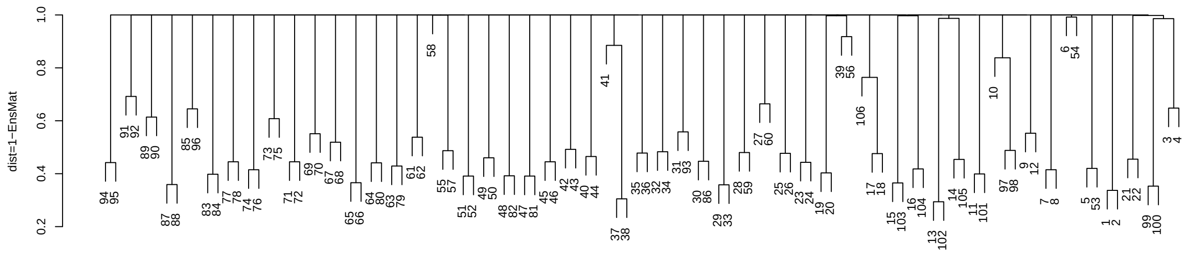

Green Trial T=0.001 Control Subject 15

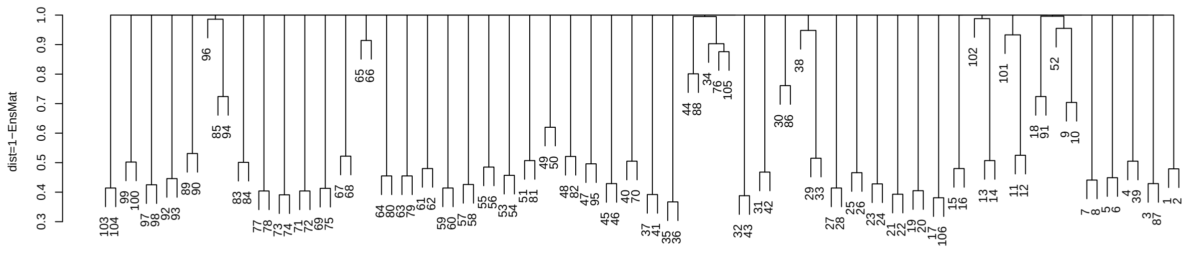

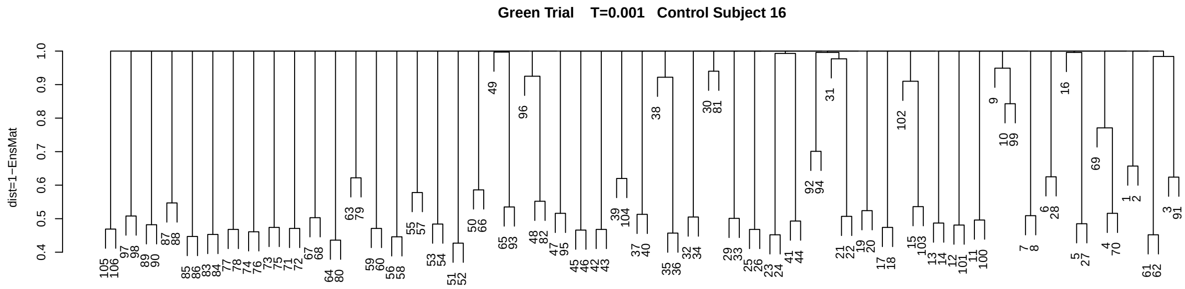
Green Trial T=0.001 Control Subject 16
dist=1−EnsMat

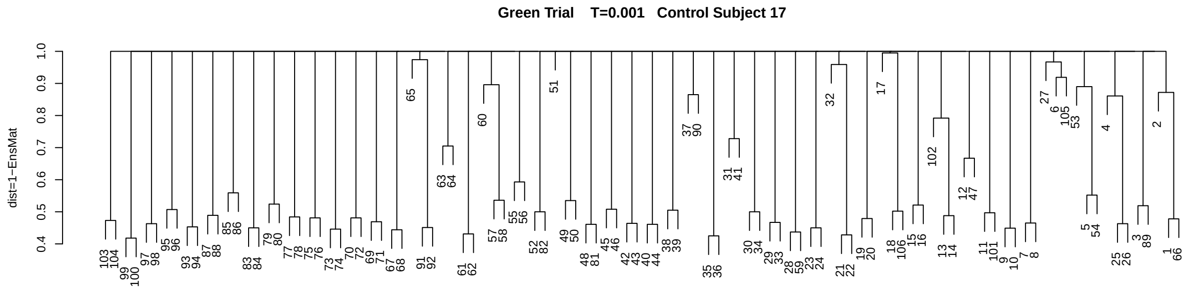
Green Trial T=0.001 Control Subject 17
dist=1−EnsMat

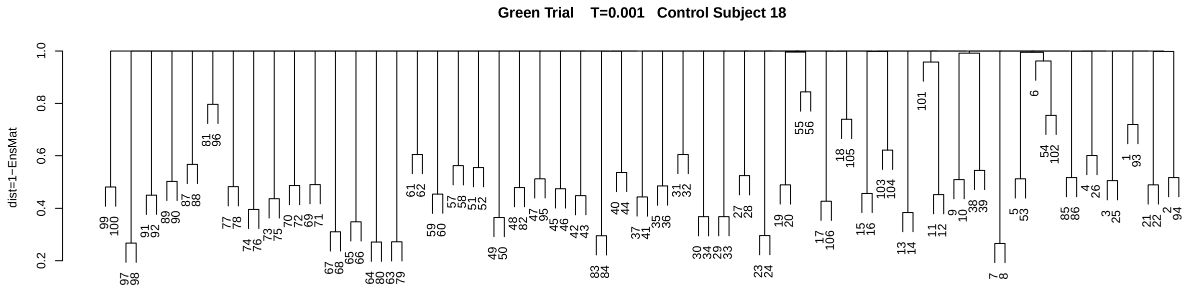
Green Trial T=0.001 Control Subject 18
dist=1−EnsMat

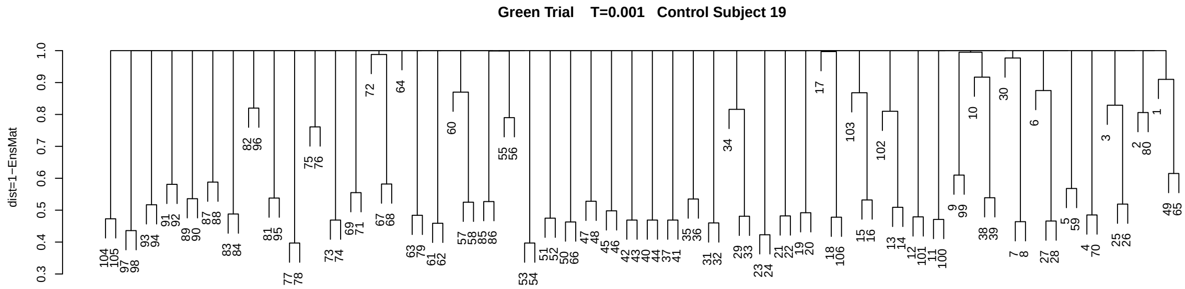
Green Trial T=0.001 Control Subject 19
dist=1−EnsMat

Green Trial T=0.001 Control Subject 20

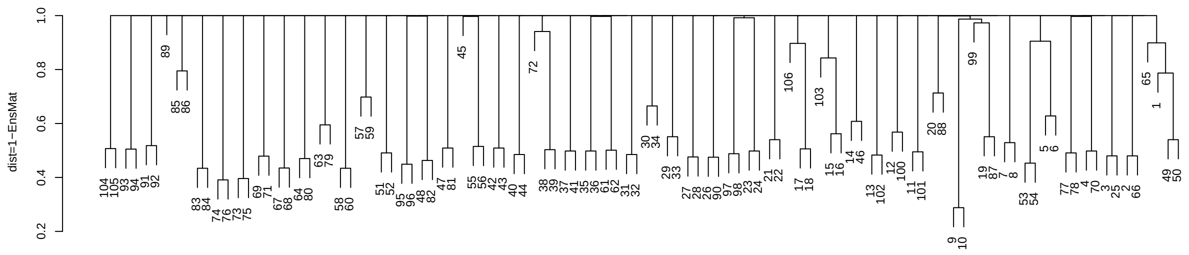

Green Trial T=0.001 Control Subject 21

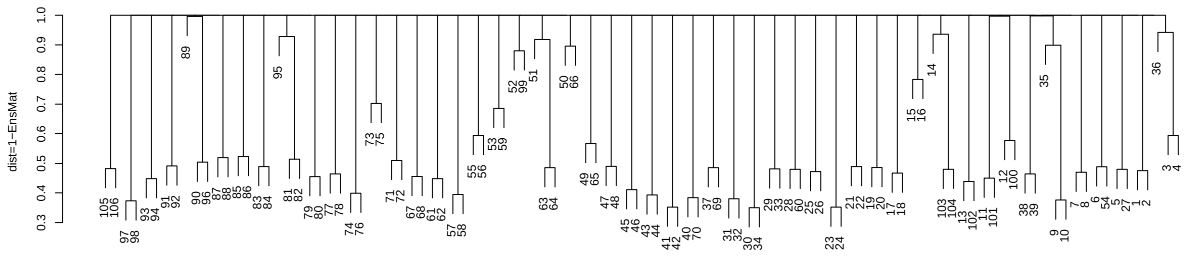

Green Trial T=0.001 Control Subject 22

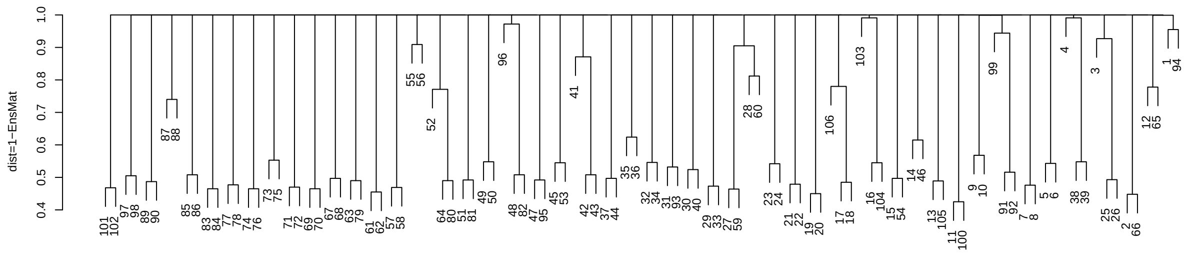

Green Trial T=0.001 Control Subject 23

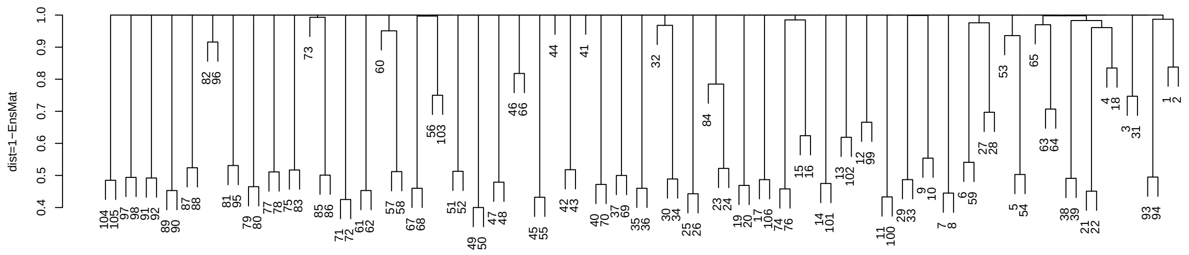

Green Trial T=0.001 Control Subject 24

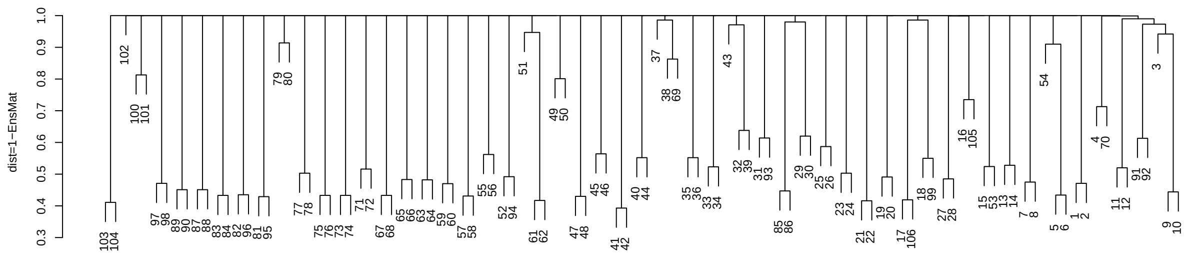

Green Trial T=0.001 Control Subject 25

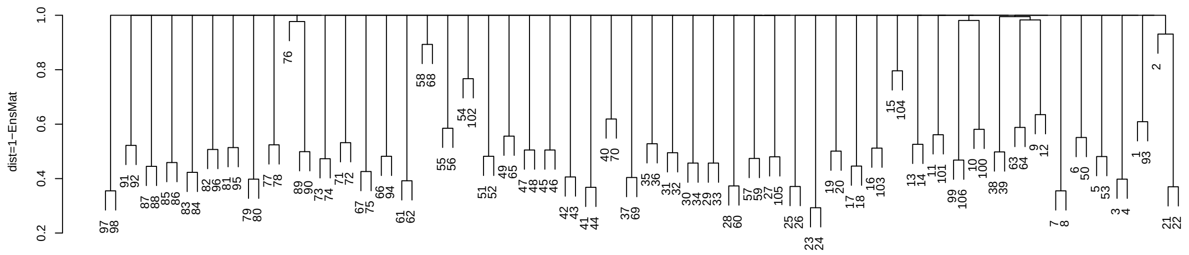

Green Trial T=0.001 Control Subject 26

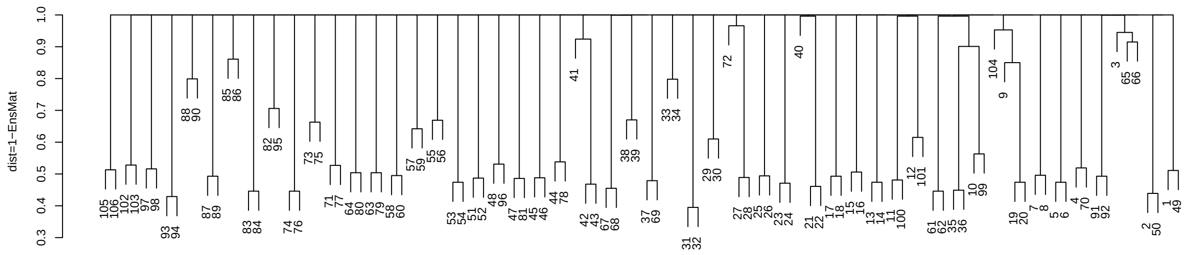

Green Trial T=0.001 Control Subject 27

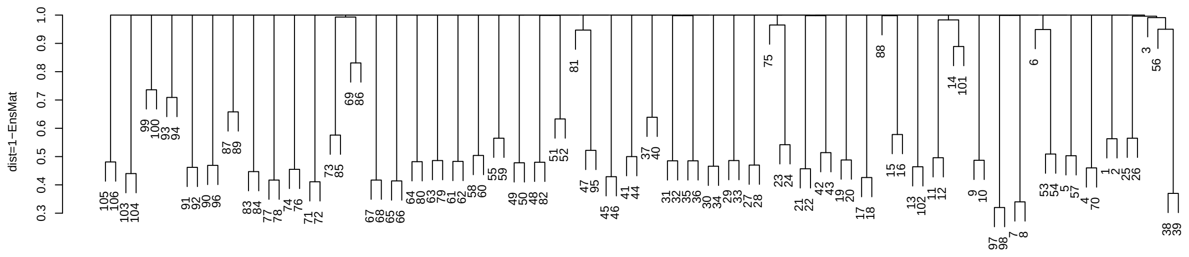

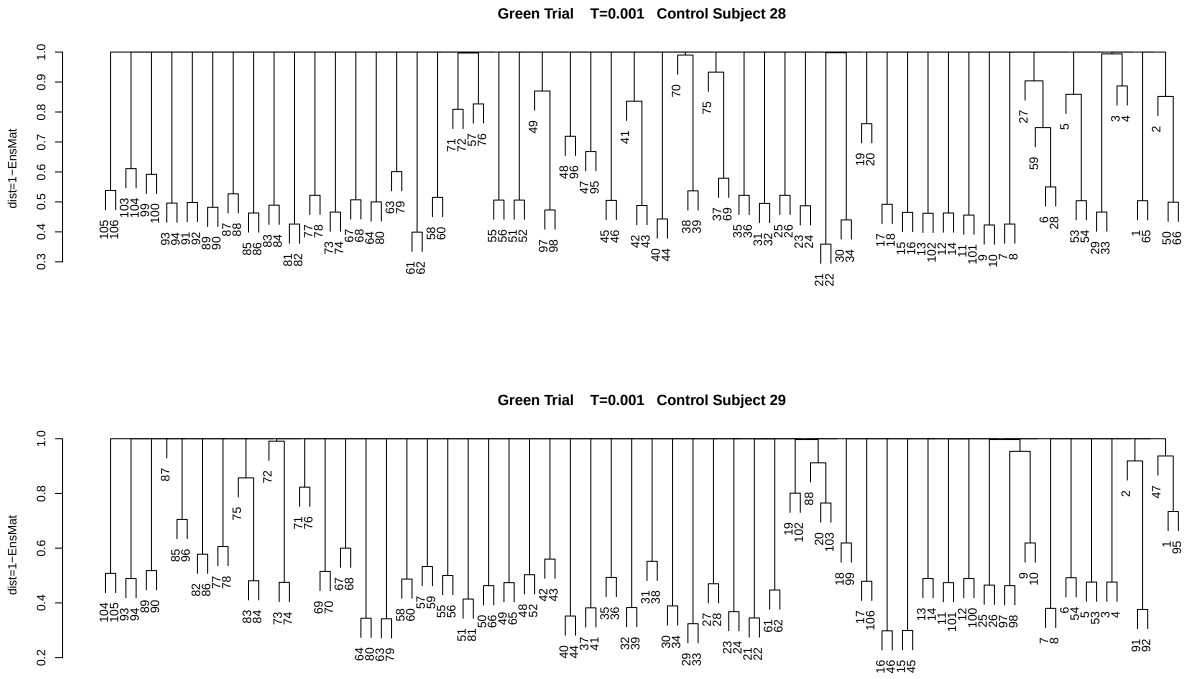

Figure 1. Hierarchical Clustering Tree of 106 ROIs for each subject in ASD group and TD group during Green Trials based on ensemble matrix (cluster-sharing probability matrix) obtained at T=0.001.
